# DESC: An Automated Strategy to Efficiently Account for Dynamic Environment Effects in Solution

**DOI:** 10.1021/acs.jctc.5c00002

**Published:** 2025-02-28

**Authors:** Albert Masip-Sánchez, Josep M. Poblet, Xavier López

**Affiliations:** Departament de Química Física i Inorgànica, 16777 Universitat Rovira i Virgili (URV), Marcel·lí Domingo 1, 43007 Tarragona, Spain

## Abstract

The properties and dynamic behavior of molecules in liquid solutions depend critically on the solvent and other species, or cosolutes, including electrolytes (if present), especially when molecular association or pairing occurs. In Quantum Mechanical (QM) calculations, the electronic structure of molecules in liquid solution is typically obtained with implicit solvent models (ISMs). However, ISMs cannot differentiate between, for example, cation types (e.g., Cs^+^ versus *n*Bu_4_N^+^), leading to limited accuracy in capturing possible solute-specific interactions. Addressing this issue in QM calculations often requires an explicit treatment of the cosolute, typically a counterion, a challenging approach due to the definition of representative cosolute positions, numerical convergence, and high computational cost for bulky species. A new computational strategy called Dynamic Environment in Solution by Clustering (DESC) is herein presented, which leverages classical Molecular Dynamics (MD) data to feed QM calculations, enabling the inclusion of counterion-specific effects with greater detail and efficiency than ISMs. DESC is particularly advantageous in cases where ion pairing/aggregation is significant, offering chemically representative QM results at a small fraction of the computational cost associated with the explicit inclusion of counterions in the model. This work presents MD data on polyoxometalate-counterion-solvent systems, introduces the philosophy behind DESC and its operational details, and applies it to polyoxometalate solutions and other relevant systems, comparing outcomes with benchmark QM/ISM calculations.

## Graphical abstract

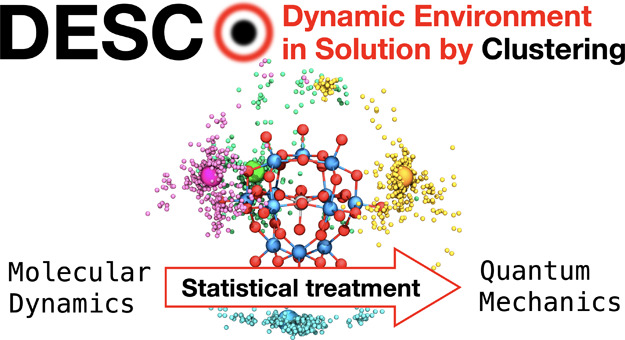

## 1 Introduction

Chemical processes mostly occur in liquid solutions, including all bioactivity in aqueous media. Consequently, the detailed chemical comprehension of solution effects, along with the appropriate application of solvent models in computational chemistry (both in classical molecular mechanics and in quantum mechanical calculations) has been subject of extensive research since its theoretical inception in the 1920s^1^ and continues to be an area of intensive study today.^2–6^

The increase of computational power, combined with more efficient algorithms, allow for the inclusion of solvent effects and other factors related to the surroundings of target molecules into calculations and simulations, primarily through the use of solvent models. This progress has dramatically improved theoretical results, such as the accurate calculation of thermodynamic properties of processes in solution.^7,8^ The well-known implicit solvation models (ISMs), such as the Conductor-like Screening Model (COSMO)^9^ and the Polarizable Continuum Model (PCM),^10,11^ are applied routinely to introduce the stabilizing effect of a liquid solution acting on some solute molecule (the *target*). The underlying idea of ISMs is to embed a molecule (the solute) within a cavity^12^ that includes the effects of the surrounding liquid solution, the theoretical treatment of which mimics that of a homogeneous polarizable continuum material. The charge distribution of the solute and the solution mutually interact via the cavity surface, inducing a mutual charge polarization. In the end, changes are induced in the electron density of the solute upon incorporation of the solvent model.

Accounting for the solution effects implicitly provides correct results in general, this approach being capable of reproducing some general features of reactive processes, including energetic aspects at a qualitative or semiquantitative level, and predicting solvation Gibbs energies. This is possible because, in many cases, the portion of the solution in contact with the target solute does not present specific localized inhomogeneities. That is, it behaves as a dielectric regular bulk that can be characterized by the few parameters of an ISM, being the solvent’s relative permittivity, ε_bulk_ and its molecular radius the most relevant. Nevertheless, this behavior can change significantly for an ionic solute because its close environment can easily include, besides solvent molecules, interacting counterions in an ion pairing fashion with no mediating solvent molecules. By applying an ISM, the system’s total charge is compensated by a set of *mirror* charges, not related to the actual nature of the counterion, and only influenced by the characteristics of the solute and by the relative permittivity of the solvent. These limitations do not permit capturing the actual effects of ion pairing onto the electronic structure of the target molecule. Consequently, regular ISMs cannot distinguish between cations (for example Li^+^, K^+^, Me_4_N^+^ or *n*Bu_4_N^+^), which can affect differently the solute properties if ion pairing takes place. Literature has shown that the cationic component of a salt in solution can have an impact on various properties, including shifts of several hundred mV in electrochemical waves.^13–15^ Of course, counterions attached to the solute’s surface can influence its reactivity and the stability of excited states,^13,16^ among other consequences. Hence, a proper computational model accounting for these interactions is mandatory for a correct description of the system characteristics.

Polyoxometalates (POMs) constitute a large family of molecular polynuclear metal-oxides, typically anionic in nature, carrying charges ranging from very low, −1 or −2, to formally very high, −15 or even greater. For this reason, they often form ionic pairs in solution, such as the widely used quaternary ammonium cations, R_4_N^+^ in organic solvents. POMs have been largely studied in our and other groups without (in the earliest stages) and with ISMs since the early 2000s.^17^ Indeed, it has been systematically shown that accounting for the effects of the target surroundings as part of the QM calculation is required for a reasonably good description of its molecular properties, among which the electron distribution via the molecular orbital shapes and energies,^18,19^ although some general features can sometimes be equally nicely reproduced within the gas phase approximation.^20^

The effect of the environment on POMs has been thoroughly reported by our group.^17,21–24^ In a recent study, we presented a combined experimental and computational investigation of a Wells-Dawson species functionalized with light-harvesting organic antennas.^13^ In liquid solution, certain electronic properties, such as excited-state lifetimes and redox potentials of the target system, exhibit a clear dependency on the solvent (CH_2_Cl_2_, MeCN, and DMF) and/or the counterions (Me_4_N^+^, Et_4_N^+^, and *n*Bu_4_N^+^). These variations determine, at the time scale of excited-state relaxations, whether a given system is a promising candidate for generating a technologically relevant charge-separated state. That QM analysis explores the effect of cation substitution on the electronic properties of the POM in solution Figure 1(left)). Given that an ISM cannot internally distinguish between cations, we defined models with explicit counterions (taken from MD simulations) in proximity of the POM for our calculations. Depending on the solvent/counterion combination, they included 4–6 medium- to large-sized organic cations, entailing long computational times, considerable human effort and, in some cases, poor numerical convergence.

**Figure 1:**
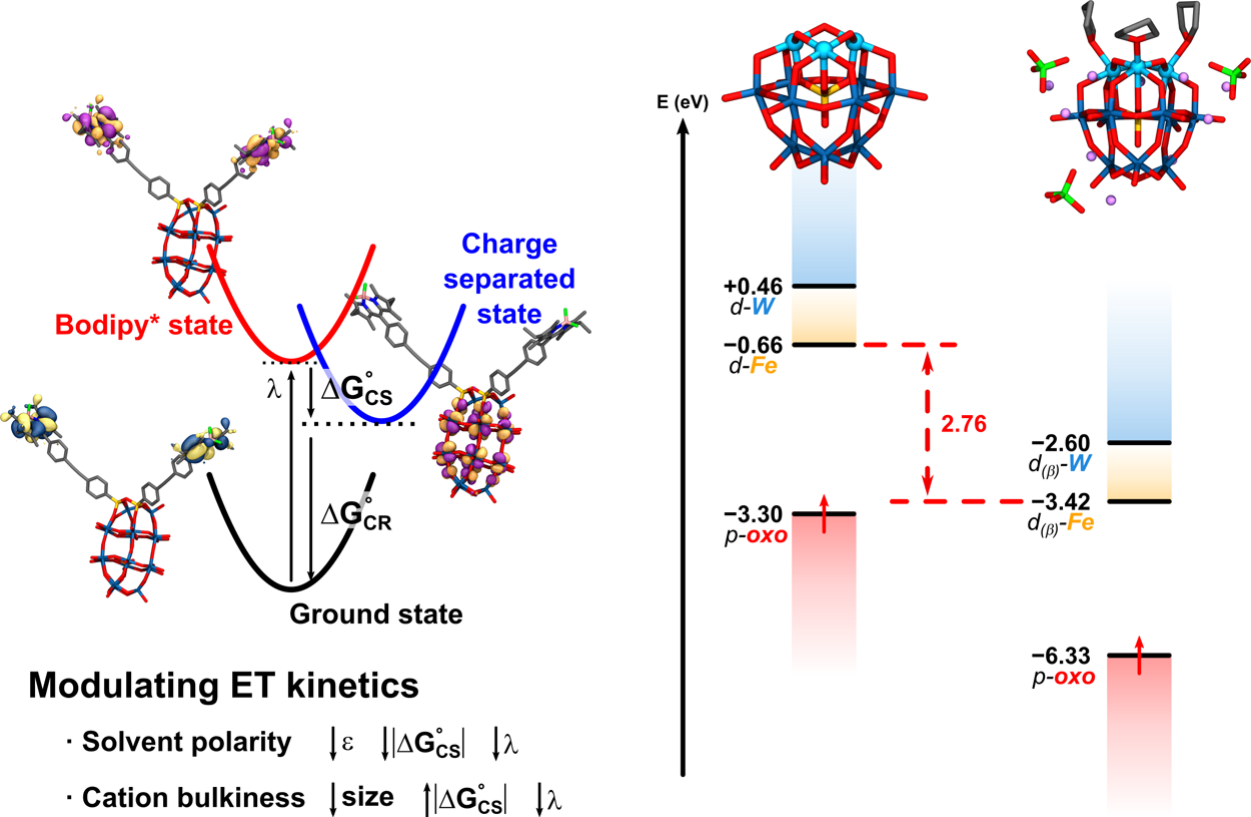
(Left) Influence of the solvent and cations on the electronic properties of a hybrid polyoxometalate.^13^ (Right) Influence of the explicit environment on the frontier molecular orbital energies of a mixed-metal polyoxometalate.^25^ In both cases, the explicit inclusion of counterions in the QM calculations was essential to rationalize the experimental observations.

Another case studied by our group involves a triiron Keggin-type POM (SiFe_3_W_9_O_37_) capable of binding and activating nitrogen with tetrahydrofuran as solvent, but only in the presence of lithium ions. The process consists of two consecutive reductions: the first one reduces the Fe^III^ centers to Fe^II^, while the second one is attributed to the catalytic activity, i.e., the formation of NH_3_ from N_2_ in the presence of a proton source. Initial MD simulations on the solution evidenced notable Li^+^-POM ion pairing. The following DFT calculations demonstrated that countercations must be explicitly included in the model to accurately reproduce the experiments. Thus, when ion pairing occurs it can play a crucial role, exerting a significant effect that cannot be described solely with an ISM model (Figure 1, right).

The incorporation of explicit ions in DFT calculations requires a detailed atomistic description of the solute environment.^26^ The most reliable in silico method to obtain such ion arrangements is through molecular dynamics (MD) simulations, which provide the trajectories of all species in solution. The next step involves selecting several snapshots (nuclear positions at different simulation times), and selecting the coordinates for the desired POM and counterions units. Finally, these are used into the corresponding DFT calculations. To ensure statistically representative results, multiple DFT runs on the selected snapshots are necessary. This methodology to include the counterion effects is, therefore, time-consuming since it requires significant manual intervention and extensive computational time. It is also important to note that processing MD trajectory data and preparing QM input files are tedious tasks that, without some level of automation, can become particularly labor-intensive and cause of error.

Besides POMs, many other systems present aggregation as herein described. Among the compounds that have indisputable technological interest are fullerenes which, at different reduction states, gain tendency to form ionic pairs in solution. Also, transition metal coordination compounds, radical organic systems, etc. are susceptible of presenting some degree of association with cosolutes.

It is worth mentioning that efforts to tackle this topic differently can be found in the literature. For example, in 2013, Matsui et al.^27^ proposed a protocol to correct the ISM-based redox potential values by adding a charge-dependent correction term for the counterions around charged target molecules. As a result, they considerably reduce the mean absolute errors but not capturing the structural features of solute–solute aggregation. More recently, an innovative study by Liu and co-workers^28^ has employed Machine Learning techniques to correct errors in redox potential calculations arising from implicit solvent models (primarily C-PCM) and explicit solvent models (using QM/MM calculations to treat the solute at the QM level and the solvent at the MM level). The authors use simple solute and solvent parameters to train an algorithm capable of mitigating errors associated with conventional methods, specifically systematic bias and large-error outliers, which are primarily attributed to the large uncertainties in the solvation free energy of charged species. Furthermore, the approach proposed by Liu et al. reduces the dependence of the results on the choice of the DFT functional, a well-documented issue extensively discussed by various authors.^29,30^

To address these challenges, we herein propose a novel strategy that combines MD and QM calculations in a highly automated and efficient manner. This approach is specifically designed to account for cosolute (counterion) effects in solution at a significantly reduced computational cost, functioning as an extension to existing ISMs. Our method becomes particularly relevant when a cosolute and the target solute exhibit some degree of aggregation, as these interactions can lead to significant differences in the local environment of the solute—and thus its electronic structure—depending on the counterion. This scenario is common in various chemical processes, including concentrated electrolyte solutions, reactivity, and ion pairing in poorly soluble species, among others. If no molecular pairing takes place, the proposed approach naturally reproduces the ISM results.

## 2 Models and Methods

### 2.1 Computational Details

#### 2.1.1 Classical Molecular Dynamics

Atomistic molecular dynamics (MD) simulations with explicit solvent molecules were performed with the GROMACS 2019.3 code^31,32^ to determine the behavior in solution of a set of POM anions together with the quaternary ammonium cations namely Me_4_N^+^, Et_4_N^+^, and nBu_4_N^+^. Simulations make use of a modified AMBER 14 force field, which has been satisfactorily employed to study the aggregation of POMs in different environments.^33^ The force field provides the potential energy of the system as the sum of bond, angle and dihedral deformation energies and nonbonding terms. The latter consist of pairwise additive 1-6-12 electrostatic and van der Waals potentials that account for interactions between atoms that are separated by more than three bonds.

Force field parameters for POMs were obtained following the procedure by López et al.^34^ using the TopoMOx code.^35^ Atomic charges to reproduce the molecular electrostatic potential (CHelpG) were obtained at the DFT level with the Gaussian 16 package^36^ using the B3LYP functional.^37^ Basis sets of double-ζ quality were used: LANL2DZ­(f) for W and a Pople-type 6-31+G­(d,p) for the rest. For the MD trajectories, cubic boxes were used with 3D-periodic boundary conditions, containing one POM anion, the number of cations required to neutralize the system and the embedding solvent for ca. 30 mM POM concentration. Solvent molecules were described by the full-atom model provided by van der Spoel and co-workers.^38^

For 1–4 van der Waals interactions we applied an interatomic distance cutoff of 14 Å, and for Coulombic interactions of 14 Å corrected for long-range electrostatics by using the particle–particle mesh Ewald (PME) summation method. All bonds were restrained by the LINCS algorithm. Production trajectories were performed on a NVT canonical ensemble during 20 ns, collecting data from the trajectories every 1 ps. Simulations were carried out at 298 K, controlling the temperature by coupling the system to a thermal bath using the velocity-rescaling algorithm. Before production runs, all systems were equilibrated by an initial 1 ns run at constant NPT to readjust the box size and a final 1 ns run at constant NVT with a relaxed solute.

#### 2.1.2 Single Point Calculations

DFT calculations were carried out with the ADF 2022 program package^39^ using the GGA BP86 exchange-correlation functional^40,41^ and a Slater basis set of TZP quality. The solvent effects were introduced via the conductor-like screening model (COSMO) with different solvents.^9,42^ The relativistic effects via the zeroth-order regular approximation (ZORA) were included. Initial structures were fully optimized, while the rest of the calculations (explicit and DESC model) were single point calculations.

In this work we put the focus on molecular orbital energies, which are typically linked to many macroscopic properties such as redox potentials or chemical reactivity, among many others. Thus, in some instances, having a more detailed and realistic picture of the nearby region of the solute is necessary for a better accuracy in the computational description of the electronic properties.

### 2.2 Target Systems

The selected target species are the Lindqvist-type isopolyanion, [W_6_O_19_]^2–^ (hereafter, L), and the Keggin-type aluminate heteropolyoxoanion, [α-AlW_12_O_40_]^5–^ (hereafter, AlK), in two reduction states: the fully oxidized and the two-electron reduced forms, with correspondingly different molecular charges. Quaternary ammonium cations of different size, that is Me_4_N^+^ (TMA), Et_4_N^+^ (TEA), and *n*Bu_4_N^+^ (TBA), were used as counterions in solution. These were simulated in two solvents with differing polarity: CH_3_CN (MeCN) and CH_2_Cl_2_ (DCM), with relative permittivities ε = 37.5 and 8.9, respectively. These combinations not only exhibit strong POM···cation ion-pairing but are also common in studies of POMs for various applications, such as catalysis and energy storage,^13,43,44^ where the solute environment is crucial. The cases analyzed represent a broad range of the chemical spectrum explored here, from small, rigid cations to large, flexible ones, displaying diverse coordination patterns depending on the POM’s charge density, size, solvent polarity, and its capacity to solvate the anionic cluster. A graphical representation of the studied systems can be seen in Figure 2.

**Figure 2:**
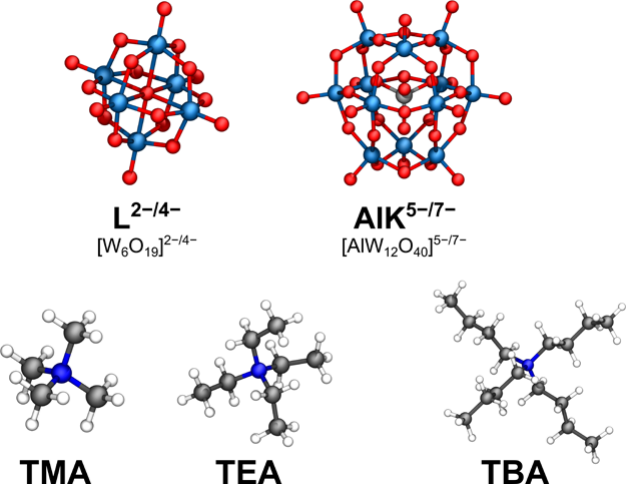
Ball-and-stick representations for the main POM (anionic, top) and counterion (cationic, bottom) species of the study. Anion–cation combinations with MeCN and DCM solvents were simulated. Color palette: W—blue, O—red, Al—silver, N—dark blue, C—gray, H—white.

## 3 DESC Methodology

### 3.1 Dynamic Environment of POMs in Solution

To obtain a detailed description of the solute environments, we conducted fully atomistic classical MD simulations in different media defined by the solvent and the counterions (see Section 2). The analysis presented here emphasizes the spatial distribution of the solvent and the nearby counterions in solutions containing a POM molecule in various reduction states. This analysis aids in understanding how the solute’s surroundings can vary depending on the counterions present and highlights the importance of accounting for this phenomenon to achieve reliable QM-based results.

Table 1 presents the most relevant parameters of the model, derived from the resulting MD trajectory: the average aggregation number, the corresponding maxima of the highest peak of the radial distribution function (RDF) (1st), and the integration distance (*I*).

**Table 1:** Relevant Data Taken from 24 MD Simulations of the Model Systems: Combinations of Two POMs, Two Reduction States, Three Countercations, and Two Solvents^a^

POM	***q***	solvent	cation	***N***	**1** **st**	***I***
L	–2	MeCN	TMA	1.04	6.370	9.480
			TEA	1.93	6.659	10.365
			TBA	1.13	6.664	9.370
	–4		TMA	4.00	6.071	8.480
			TEA	3.99	6.071	7.777
			TBA	1.71	6.483	9.297
	–2	DCM	TMA	1.99	6.269	9.479
			TEA	1.93	6.577	9.690
			TBA	1.72	6.761	10.267
	–4		TMA	4.00	6.076	7.884
			TEA	4.00	6.056	8.158
			TBA	2.99	6.264	9.171
AlK	–5	MeCN	TMA	4.33	6.976	8.582
			TEA	4.99	7.070	9.978
			TBA	3.71	8.057	10.659
	–7		TMA	7.00	6.960	8.362
			TEA	7.00	6.975	9.383
			TBA	3.36	7.955	10.857
	–5	DCM	TMA	5.00	6.965	9.971
			TEA	5.00	7.078	8.684
			TBA	4.19	7.860	10.964
	–7		TMA	7.00	6.955	8.356
			TEA	7.00	6.965	9.069
			TBA	5.92	7.678	10.287

a The distance of the first peak maximum (1st) and the integration distance (*I*), both in Å, and the averaged coordination number (*N*) are given. Data taken from the last 10 ns of the trajectory, sampled every 1 ps.

This exhaustive table lists the relevant data obtained from the aforementioned MD simulations. Key observations on POM···cation ion-pairing across the analyzed systems can be summarized as follows:

- Smaller cations consistently exhibit closer and stronger aggregation due to their size and hardness: **TMA**(**N, 1** ^**st**^, **I**) > **TBA**(**N, 1** ^**st**^, **I**).
- In more polar media, such as MeCN, POMs and cations are better solvated, resulting in fewer ion pairs: **DCM**(**N, 1** ^**st**^, **I**) > **MeCN**(**N, 1** ^**st**^, **I**).
- POM···cation association reaches saturation more rapidly for larger cations, which may eventually interfere with one another sterically or electrostatically.

These observations are well established within the scientific community and are consistent with previous studies^13,33,45,46^ and with the findings presented in this work. Nevertheless, their significance remains undiminished, as they provide an exceptionally detailed description of counterion positioning around the *target* solute. Additionally, they enable precise tracking, at the angstrom scale, of the evolution of both ionic pairs and solvent molecules throughout the solution. Two examples of the simulation results are shown in Figure S1: the (TMA)_5_AlK salt in MeCN and the (TBA)_4_L salt in DCM, which illustrate the observations discussed above.

The perspective on the dynamic environment of a solute (in this case, a POM anion), which varies significantly based on numerous parameters, provides a foundation for developing an alternative computational methodology. By directly extracting data from MD trajectories in an ordered and automated way, we construct a model that incorporates this information in a fast, straightforward, and user-friendly manner, integrating it into routine QM calculations to account for the effect of the environment on the target solute properties.

### 3.2 Influence of the Explicit Environment on the Electronic Structure of the Solute

With a detailed description of the environment, the effect of counterions on the POM was analyzed with the traditional methodology: selecting *N* random MD snapshots (in this case, five) representing aggregation, obtaining the respective atomic coordinates, and performing QM (specifically, DFT) calculations. Then, the electronic properties were determined by averaging the results of the five DFT runs. A key point to note is that, in cases where the average aggregation values for the selected snapshots are not integers, and straightforward rounding is not feasible (i.e., values between X.22 and X.78, where *X* ∈ N ), the average was weighted according to the relative frequency of the observed aggregation values:

$$\begin{array}{c} E_{i}=w_{a}\cdot E_{i}(N_{a})+w_{b}\cdot E_{i}(N_{b})\\ w_{a}+w_{b}=1 \end{array}$$ 1

In eq 1, *E*_*i*_ denotes a property (e.g., total energy or MO energy), and *a* and *b* are the subscripts of the two nearest integer values to the actual fractional aggregation number.

It should be noted that when we refer to environment effects, we are specifically addressing the influence of explicit counterions. Solvent effects are incorporated via the ISM (COSMO in this study). This strategy has been successfully employed in previous studies.^13,25,47^ Herein, special attention is given to molecular orbital energies, as these are often correlated with macroscopic properties such as redox potentials, spectroscopic properties, and chemical reactivity, among others.

Figures 3, S2, and S3 clearly highlight the significant role of explicit cations in the systems analyzed. It is evident that the molecular orbital energies can vary considerably depending on the cation being the cosolute. Consequently, taking the frontier orbitals as representative of the effects taking place on the electronic structure, the range of absolute energies computed for the highest occupied molecular orbital (HOMO) and lowest unoccupied molecular orbital (LUMO) is substantial. For instance, in DCM, the HOMO of the oxidized AlK system shifts from −6.14 to −6.71 eV when TBA is replaced by TMA, showing a difference of over half an eV. In contrast, the corresponding HOMO energy at the COSMO level is −5.51 eV. In MeCN (Figure S3), the range of energies is narrower but remains significant; for the oxidized AlK system with TMA cations, the LUMO is found at −4.14 eV, while the pure COSMO calculation gives −3.86 eV. Lastly, in the reduced AlK system in DCM (Figure S2) with TMA, the HOMO and LUMO lie at −3.19 and −2.94 eV, respectively, while the COSMO values are −1.68 and −1.45 eV. For systems containing *L* (reduced system in Figures S2 and S3), the observations are similar. The discrepancies in orbital energies are, in some cases, greater than 1.5 eV, rendering pure ISM results unreliable in systems where aggregation is present.

**Figure 3:**
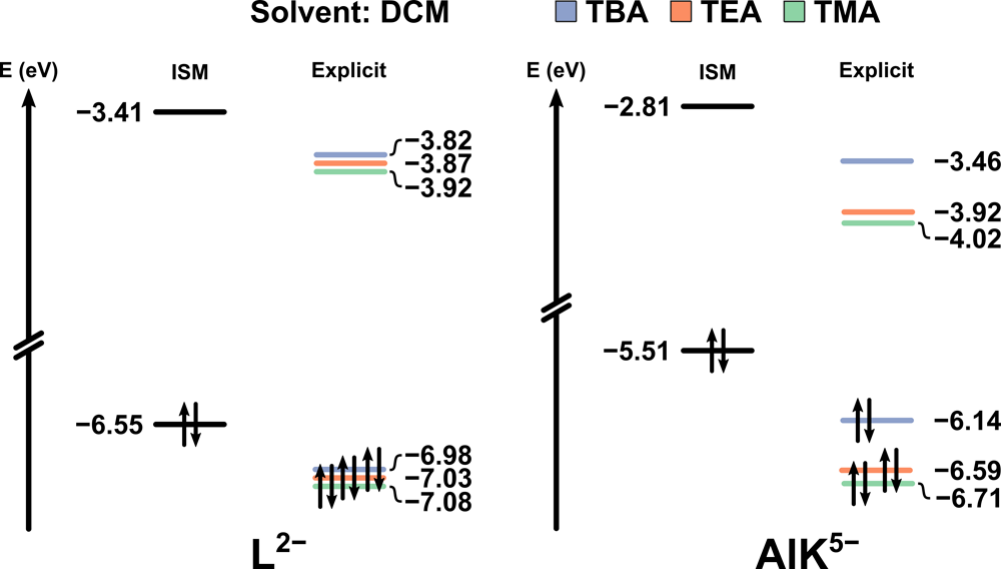
Molecular orbital diagram for L and AlK salts of TMA, TEA, and TBA in fully oxidized state, in DCM calculated following two methodologies: ISM (COSMO, ε = 8.9) and Explicit+ISM (average of 5 snapshots).

It is clear that when a pure ISM is used to simulate solution effects, the calculations cannot distinguish between different cations. Moreover, the pure COSMO results hardly resemble any of the explicit models used as benchmarks. However, the HOMO–LUMO gaps across all explicitly treated cases are quite similar (differing by approximately 20–30 meV), suggesting that certain properties dependent on these gaps may still be reasonably reproduced by the ordinary solute + COSMO approach. It is worth mentioning that the electronic energies obtained with COSMO are not always the least stable, particularly in solvents with high relative permittivity (such as MeCN). This aligns with expectations for ions in solution, where electrostatic interactions are influenced by 1/ε. Conversely, in DCM, the electronic structure description provided by COSMO alone is poor and deviates considerably from that of the explicit aggregated solute model.

The next step is how can we combine the environmental effects introduced by COSMO with those of the aggregated cosolute? At this point, it is important to stress that a given new strategy must be computationally cheaper and more efficient than the method of explicit cations mentioned above.

### 3.3 New Strategy to Account for the Environment in Solution: DESC

When analyzing the differences between an ISM calculation and a calculation with explicit counterions, two primary factors can be distinguished:

- Electrostatic effects: The cosolute surrounding the target molecule may be a charged species that influences its electronic structure. Additionally, since the solvent cavity is generated by point charges that model ISM polarization, the presence of an adjacent species alters the ISM solvent cavity itself, leading to modified charge distributions in certain regions of the cavity and, consequently, affecting solvation.
- Steric effects: Cosolutes aggregated around the solute occupy physical space, reducing ISM interactions with that part of the target molecule.

With the results from the previous section and these foundational considerations in mind, we developed the new methodology coined Dynamic Environment in Solution by Clustering (DESC), which is described in detail below. The computational strategy employed by DESC has been implemented in a Python code^48^ that, given a MD trajectory in PDB (Protein Data Bank) format and a simple input file with basic system information, analyzes the MD simulation and generates an ADF input file ready for submission, thus incorporating the effects of aggregated ions on the solute. This model is broadly applicable, with no restrictions regarding molecular charge, nature or number of constituent molecules, or solvent type. The use of ADF software is not limiting; the concepts behind DESC are adaptable to other computational software. However, at this stage, testing has been conducted exclusively with the ADF program. The DESC code can be easily used by installing it from the GitHub platform (https://github.com/qcgurv/DESC).

One of the most unique aspects of DESC is how it accounts for the counterion effects by introducing them through: (1) a cloud of point charges positioned at the locations of counterions aggregated during the MD simulation, and (2) a set of ghost atoms with explicit spherical volumes simulating the presence of cations aggregated to the target solute, placed at the most representative positions. With appropriately selected radius and charge parameters (described below), this methodology replicates the effects of a calculation with explicit counterions without double-counting their impact on the ISM. While COSMO already implicitly introduces the average effect of counterions contained in the bulk, the introduction of ghost atoms with volume, which restrict the ISM from reaching certain solute regions while providing an adjusted surrounding charge distribution, reproduces the localized effects of explicit counterions.

Previous to applying DESC, it is essential that the target solute retains consistent orientation (both rotational and translational) with respect to the other molecules surrounding it throughout the MD trajectory. This consistency ensures that all frames processed by DESC are equivalent and comparable, thereby accurately reproducing the cosolute environment. Trajectories can be easily converted using specific software like GROMACS, Python libraries such as MDAnalysis,^49,50^ or VMD scripts.^51^ These algorithms employ a frame from the MD simulation as a reference, minimizing the root-mean-square deviation (RMSD) between frames by fixing one residue (herein the target molecule) and adjusting the rest accordingly. Figure S4 shows two example snapshots from a simulation of (TBA)_7_AlK in MeCN, before and after reorientation. Effectively, this process aligns the classical trajectory by fixing the position of one residue (the target solute, i.e., the POM) while repositioning everything else, including the simulation box. Original energies and interactions remain unaltered during this process.

The complete DESC workflow is illustrated in Figure 4. The following section will detail each step to explain how we transform a MD trajectory into a ready-to-run QM input file.

**Figure 4:**
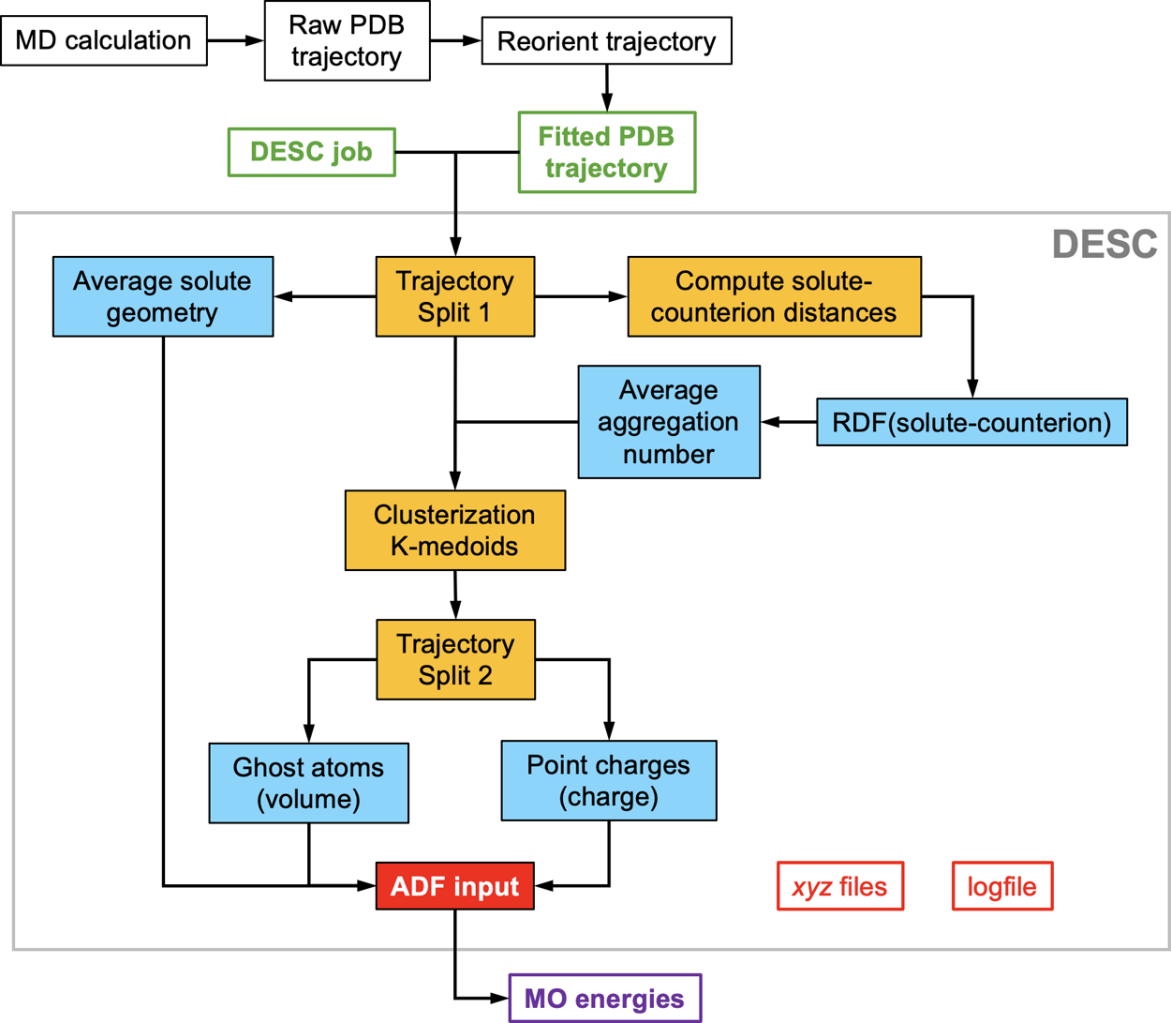
Schematic view of the main data treatment associated with DESC. Taking the raw PDB data from a MD run, an initial transformation must be done to generate the fitted PDB which, together with a job-DESC input file, feed the program (green boxes). A simplification of the DESC workflow is represented inside the thin box, with tasks and outputs represented in orange and blue.

#### 3.3.1 Input File

Once the trajectory has been reoriented based on the position of the solute, the next requirement for DESC to function is basic information about the system. To maintain the simplicity of the method, the input file requires only:

- The name of the trajectory file (in PDB format)
- The number of frames to be analyzed
- The name of the solute residue
- The name of the aggregated cosolute residue, its most representative atom, and its formal charge

An example of the input file for a TMA-AlK system in MeCN is provided in the SI.

#### 3.3.2 Splitting Frames

Even though all frames in a dynamic simulation are equally important, not all are necessary in this approach; only a selection is taken and processed. In the “standard” method for explicit QM calculations, the first step implies selecting a collection of well-spaced snapshots and, then, QM calculations are run. Postprocessing of these results generate an average of the electronic property under study, assumed representative. Therefore, in a small to medium trajectory containing 10,000–50,000 frames (10–50 ns), at least 5–10 snapshots (and their QM runs) should be involved, despite the sizable computational and human cost associated. Instead, DESC extracts first a large number *N* of frames from the MD trajectory to work with. A relatively large *N* is crucial to ensure good representativeness of the DESC result. We have determined that *N* = 400 for this initial split is largely sufficient. Using a lot less frames may yield a poor description of the solute environment, while a lot more would slow down the analysis without improvement of the results. From this initial split the radial distribution function (RDF, *g*(*r*)) between the centers of mass (COMs) of the solute and the counterions is calculated and, then, the associated average number of ions aggregated around the solute and their distances. Therefore, the number of frames is critical for achieving a well-resolved RDF (see Figure S5 for further information).

From the set of 400 frames selected, a subset of 100 frames is taken (either one every four consecutive frames or randomly, the result has proven to be consistent), from which the charge distribution surrounding the target molecule is captured and used as input data for the last QM calculation step. This selection influences the ease of convergence in the self-consistent field (SCF) procedure during the QM calculation, its speed, and how accurately the ionic environment of the solute is described, depending on the number of point charges included. The rationale for selecting 100 frames is illustrated in Figure S6, which shows how the energy of the frontier molecular orbitals of an L^2–^ evolves based on the number of frames in this second split. Importantly, if only point charges are included, the orbital energies remain different from the ones of the snapshot.

#### 3.3.3 Average Solute Geometry

Another feature of DESC is that it provides the user with a nearly optimized structure of the target solute for carrying out QM calculations. From the first split, the 400 solute structures are collected and an average structure is generated. This calculation benefits from the initial reorientation, where all solute structures analyzed by DESC share the same orientation and minimal internal RMSD. The resulting structure is considered nearly optimized because it is derived from a dynamic simulation where, in principle, the solute geometry is optimized, and the force field applied does not significantly distort the molecule.

An example is provided for AlK^5–^ in MeCN, where the electronic energy difference (Δ*E*) between the DFT-optimized structure and the DESC-generated structure is 1.96 kcal mol^–1^, with the frontier molecular orbitals showing a mean absolute error (MAE) of 20 meV (−6.56 vs −6.54 eV for the HOMO, and −3.86 vs −3.83 eV for the LUMO in the optimized vs DESC structures, respectively). Regarding molecular structure, the RMSD between the DFT-optimized and DESC-generated structures is 0.0034 Å, supporting the consistency of the electronic parameters. Reflecting this low RMSD, Table S1 shows the average bond distances and their deviations for the various bond types in AlK in both the optimized and DESC-generated structures. This feature can be highly advantageous for DESC applications, simplifying the study of environmental effects on solute reactivity or spectroscopy by assuming the solute structure is effectively “optimized.”

#### 3.3.4 Automated Analysis of the RDF

From the previously described solute-counterions RDF, whose generation and analysis are incorporated into DESC without further user input, the peak integration distance and the average number of counterions around the solute are determined. Prior to this, the *g*(*r*) function is smoothed using a Savitzky-Golay filter, which preserves peaks and curvatures by fitting a low-degree polynomial to a series of data points and then calculating the smoothed value of the signal. This filter is implemented in the code via the SciPy library,^52^ which also enables calculation of the function’s first derivative. With these tools and the SciPy’s *find_peaks* function, peak maxima (*find_peaks*) and minima (when the first derivative is zero and changes from negative to positive) are identified to establish the integration distance for each peak. If multiple peaks are present, the integration distance of the peak farthest from the solute is used. The robustness of this method was tested and confirmed during code development.

When solute and counterions do not present aggregation, the RDFs may show distant, weak peaks or integration distances much further than typically considered to be the coordination sphere of the solute. Since DESC can be applied to any simulation, regardless of the presence of ion pairs, an aggregation cutoff distance to prevent these spurious peaks was added. Thus, if a peak in the RDF is found not accomplishing this criterion, it is discarded and no aggregation is considered. The cutoff distance is system-dependent and is calculated as

$$D_{\mathrm{cutoff}}=\left(\frac{d_{\max,\mathrm{solute}}}{2}+1.5\right)+(1.5\times R_{g,\mathrm{counterion}}^{v})$$ 2

where *d*_max,solute_/2 is half the largest interatomic distance within the solute, and *R*_g_^*v*^ is the radius of gyration of the counterion, explained in detail in a later section. The interatomic distance is increased by 1.5 Å to approximate a sufficiently generic “effective van der Waals radius.” Additionally, *R*_g_^*v*^ is scaled by a factor of 1.5 to ensure that *D*_cutoff_ encompasses an adequate pairing distance. Although this distance may seem excessive or lack physical meaning, its purpose is to prevent unrealistic interpretations of the RDF that could lead to inexistent aggregation.

An example of RDF calculated by DESC is shown in Figure 5, which highlights the original RDF, the smoothed RDF, the peak maximum, the first derivative function, and the integration distance.

**Figure 5:**
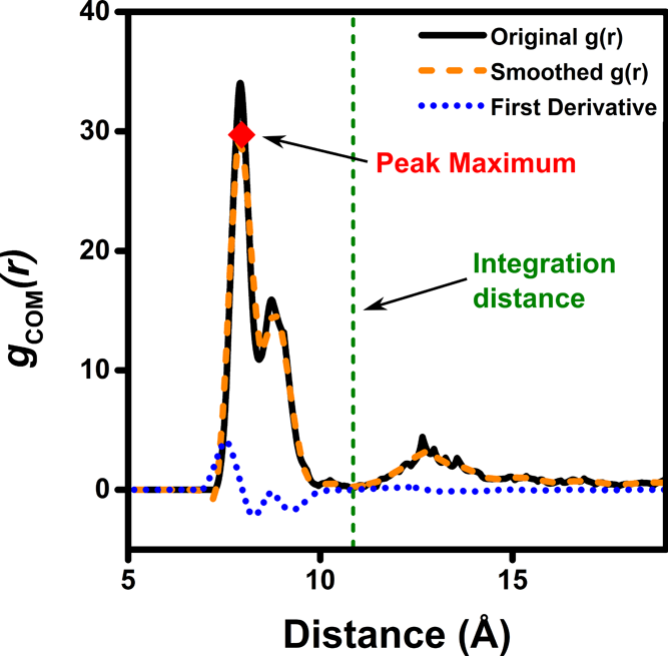
Radial distribution function and associated features, calculated by the DESC code.

#### 3.3.5 Clustering of Counterions’ Positions

At this point, DESC has collected the solute structural data and the associated distribution of aggregated ions, represented as points located at the centers of mass of the explicit cosolute molecules (or, alternatively, at the most representative atoms, such as nitrogen atoms for *n*R_4_N^+^). The next step is to group these points by spatial proximity argument(s) making *clusters* of points. This step applies a clustering method, specifically the well-known K-Medoids algorithm.^53,54^ The choice of clustering algorithm is based on the need to find as many clusters as the RDF analysis has previously identified as aggregated counterions, (*N*). Among clustering algorithms that depend on a set number of clusters (such as K-Means, agglomerative clustering, etc.), the selection of K-Medoids is not deliberate. Next, once clusters have been generated, a special point per cluster is identified and, hence, converted into a ghost atom (with no charge and with volume). While similar to K-Means, K-Medoids is applied by DESC. It minimizes the sum of the distances between each point and the medoid of its cluster, rather than the sum-of-squares within the cluster. Unlike a centroid, at the end of the procedure one medoid is identified: an existing point within the original data set, not an averaged location that may fall outside the actual collected data. Figure 6 displays the clustering results for the system (TBA)_5_AlK in CH_2_Cl_2_ (*N* = 4.19), with four clusters differentiated by color. The medoid of each cluster is highlighted with a larger sphere and will ultimately be treated as a ghost atom in the final QM calculation.

**Figure 6:**
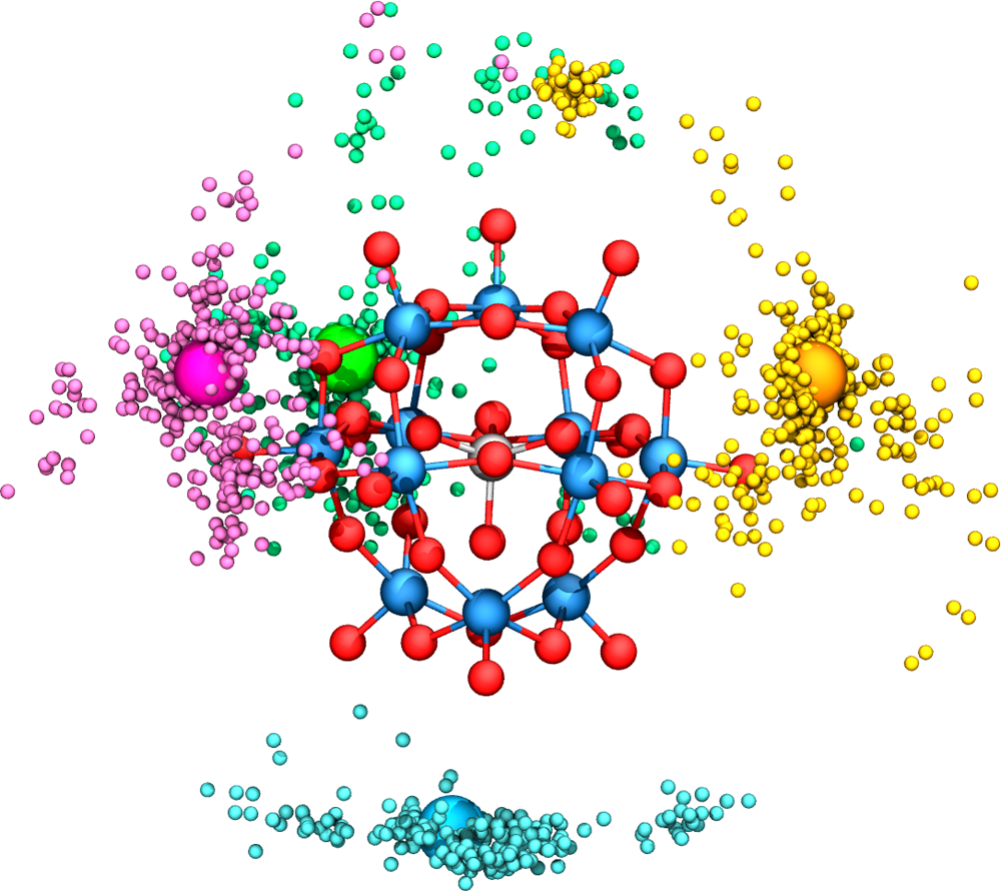
Representation of the four clusters of counterions generated by DESC (colored fuchsia, green, orange, and blue) for (TBA)_5_AlK in CH_2_Cl_2_. The four corresponding medoids are highlighted as larger spheres.

The average aggregation number obtained by DESC may be fractional. An interesting feature of DESC, similar to the explicit approach for including counterions, is that when the value obtained falls between X.22 and X.78 (*X* ∈ N ), the clustering algorithm is applied twice, once for each of the two nearest integers (*X* and *X* + 1), generating two QM input files, one for each integer. Subsequently, the electronic properties obtained from the final QM calculations are weighted according to eq 1. These weights are provided to the user in the DESC logfile.

With these components defined, we can precisely identify ghost atoms and point charges within the model based on the RDF analysis. The ghost atoms correspond to the medoids, representing the statistically *N* most representative positions of the ion groups (clusters) near the solute. The point charges are the remaining points lying within integration distance. We now illustrate the treatment applied to each component.

### 3.4 Ghost Atoms and Point Charges

#### 3.4.1 Ghost Atoms: Volume-Weighted Radius of Gyration

The role of ghost atoms and how they are created is a major innovation in this model. Currently, computational chemistry programs use spherical atomic surfaces to create the cavity surrounding molecules to simulate the ISM. This approach naturally extends to using spherical volumes for ghost atoms in DESC, regardless of the morphology of the molecule they represent. The question then arises: what radius best characterizes the residue represented by the ghost atoms? Various metrics can describe a molecular shape, such as the distance from the COM to the farthest point, the sum of van der Waals radii, or the radius of the smallest sphere encompassing the entire molecule, among others. There is no definitive answer as to which is ideal; the choice largely depends on the context and the goal of the chosen approximation. To best represent the associated counterion treated as a ghost ion with volume, we have determined that the volume-weighted radius of gyration (denoted as *R*_g_^*v*^) is the most suitable descriptor for defining the volume of each ghost atom:

$$R_{g}^{v}=\sqrt{\frac{\sum_{i}v_{i}(r_{i}-r_{\mathrm{COM}}{)}^{2}}{\sum_{i}v_{i}}}$$ 3

In eq 3, summations run over all the atoms in the molecule, *v*_*i*_ is the van der Waals volume of the *i*th atom, *r*_*i*_ is the position vector of the *i*th atom, and *r*_COM_ is the position vector of the molecule’s COM. Here, the van der Waals radii are consistent with those used by COSMO in the ADF program, derived by dividing the MM3 method radii calculated by Allinger^55^ by 1.2.

The radius of gyration provides an indication of molecular size, accounting for shape, flexibility, and conformation by representing the root-mean-square (RMS) distance of each atom from the COM. Traditionally, in polymer chemistry, each RMS distance is weighted by atomic mass. For our purposes (defining the volume occupied by a molecule as a single point at its COM), weighting each RMS distance by van der Waals volume is more suitable. DESC calculates the *R*_g_^*v*^ of each target molecule in every selected frame of each trajectory and internally computes the average, that is, it determines the *R*_g_^*v*^ directly from the simulation, thus accounting for the flexibility and deformation of the large counterions aggregated with the solute, implicitly including the effect of the solvent. This significant feature distinguishes DESC from other models. The volume of the ghost atoms prevents the ISM from forming a cavity around those regions of the solute, thereby avoiding over- or under-stabilization effects from the solvent. Figure 7 shows how DESC assigns volumes to ghost atoms, modifying the cavity around the solute to mimic the explicit model. This approach also provides a straightforward way to distinguish between counterions. The ghost atoms, however, are neutral. Although this may seem counterintuitive, particularly when the coordinated molecules are ions, the primary role of the ghost atoms in this model is to occupy the physical space occupied by the aggregated cosolute, as the explicit model does. The electrostatic influence of the surrounding ions, as explained below, is entirely provided by the cloud of point charges derived from the selected MD frames.

**Figure 7:**
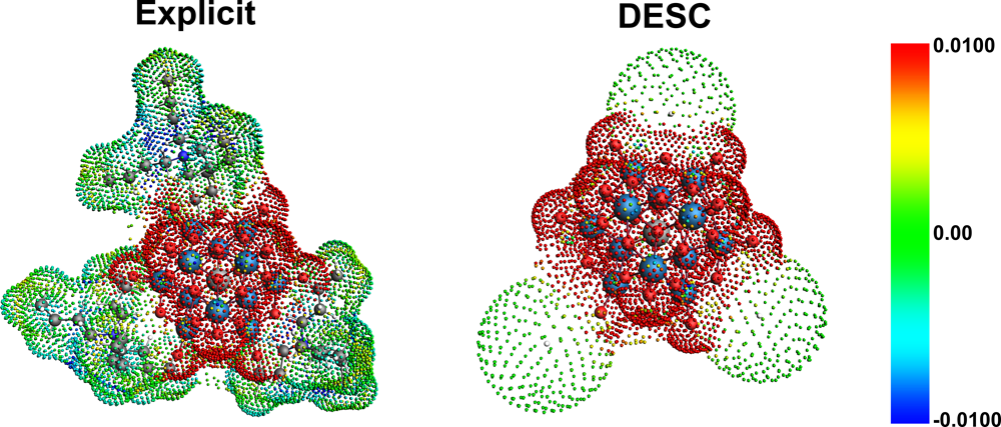
Comparison between COSMO charge density surfaces for the explicit system (TBA)_7_AlK (where *N* = 3) and the corresponding DESC approach. Despite the differences in shape between the TBA cavities and the spherical ghost positions obtained via clustering, the amount of solvent excluded from the solute surface is comparable. A color bar indicating charge density is included.

DESC automatically generates an ADF-like ghost atoms input block, **Gh.H**, that includes the *xyz* coordinates previously identified as medoids. For the SCF calculation, the ghost atom is arbitrarily a pseudohydrogen atom with zero nuclear charge and minimal basis set, a choice not affecting the electronic structure of the solute as it contributes nothing beyond occupying space. An example of the ATOMS block for a (TMA)_4_L system, where *N* = 4, can be found in the SI.

#### 3.4.2 Point Charges: Fitted Point Charges

If we assert that there are countless ways to estimate the ill-defined radius (or volume) of a molecule, attempting to mimic the electrostatic effects of an ionic cosolute with a volumeless point charge is equally challenging. During the development of this method we found that, to achieve a realistic effect on the solute, these point charges should not be equal to the formal charge of the ions that they represent (further details in the SI). Instead, these charges must be significantly lower. This adjustment is necessary due to a combination of effects involving the surrounding solvent, which polarizes the charge distribution based on its relative permittivity, the internal structure of the cosolute and its electrostatic self-screening, and interactions with neighboring ions (mainly repulsion with co-ions and attraction to other species). Altogether, these factors scale the intensity of the electrostatic interaction between the solute and the point charges in a complex manner. Nevertheless, we have parametrized the following simple equation using the systems listed in Table 1, which effectively captures the screening effect on each point charge in a straightforward manner:

$$q_{\mathrm{fitt},i}=q_{\mathrm{formal}}\frac{\left\langle N_{\mathrm{ion}}\right\rangle }{\sum_{i}N_{i}}\left(0.025+\frac{1}{\varepsilon_{\mathrm{bulk}}}\right)$$ 4

where *q*_formal_ is the formal charge of the ion (+1 for *n*R_4_N^+^), *N*_ion_ is the average coordination number of ions around the solute, ∑_*i*_ *N*_*i*_ is the total number of point charges determined by DESC, and the term in parentheses is an empirically fitted correction factor.

The above expression indicates that solvents with higher relative permittivity reduce the fitted effective charges *q*_Fitt,*i*_. This effect arises primarily because solvents with high relative permittivity can more extensively polarize the cosolute’s charge distribution, thereby diminishing the intensity of the charge experienced by the solute. Interestingly, DESC automatically detects the solvent used in the MD run (from the input PDB file) and assigns the appropriate permittivity constant for internal data processing. The solvent is not specified by the user in the DESC input; rather, it is identified by locating the most abundant residue in the PDB file, processing one of these residues to construct its molecular formula, and comparing it to an extensive internal solvent library to obtain the permittivity constant. If the solvent is not found in the library, or if the formula processing fails, the user is prompted to enter the solvent’s name and relative permittivity, which are then used to continue DESC’s calculations.

For the set of point charges (one of the main DESC outputs) the code generates a *xyz* file and also incorporates an ADF-like input block containing the *xyz* coordinates of the nonmedoids points, along with a column of charges corresponding to the *q*_Fitt_ value in atomic charge units. The number of elements in this list varies depending on the degree of ion-solute aggregation. All information generated by DESC is included in a ready-to-run ADF input file. An example of the point charge block in ADF format is provided in the SI.

#### 3.4.3 Combining Sterics and Electrostatics

Before discussing the performance of the DESC model, it is essential to emphasize the importance of the two key components of this approach: the distribution of fitted point charges and the neutral ghost atoms.

Using the (TMA)_7_AlK system in MeCN as an example, where *N* = 7, Figure 8 illustrates the contribution of each component to the electronic structure of the salt, spanning from the highly destabilized gas-phase approach to the DESC model, in excellent agreement with the benchmark explicit model results. This demonstrates not only the accuracy of DESC but also the individual impact of the two main components of this model. Applying ghost atoms only, the ISM surface has reduced contact with the solute, leading to destabilized orbital energies compared to the ISM-only model. Only when the cloud of fitted point charges is added to the final QM calculation do the energies align with those obtained using the explicit model, significantly improving the ISM-only results.

**Figure 8:**
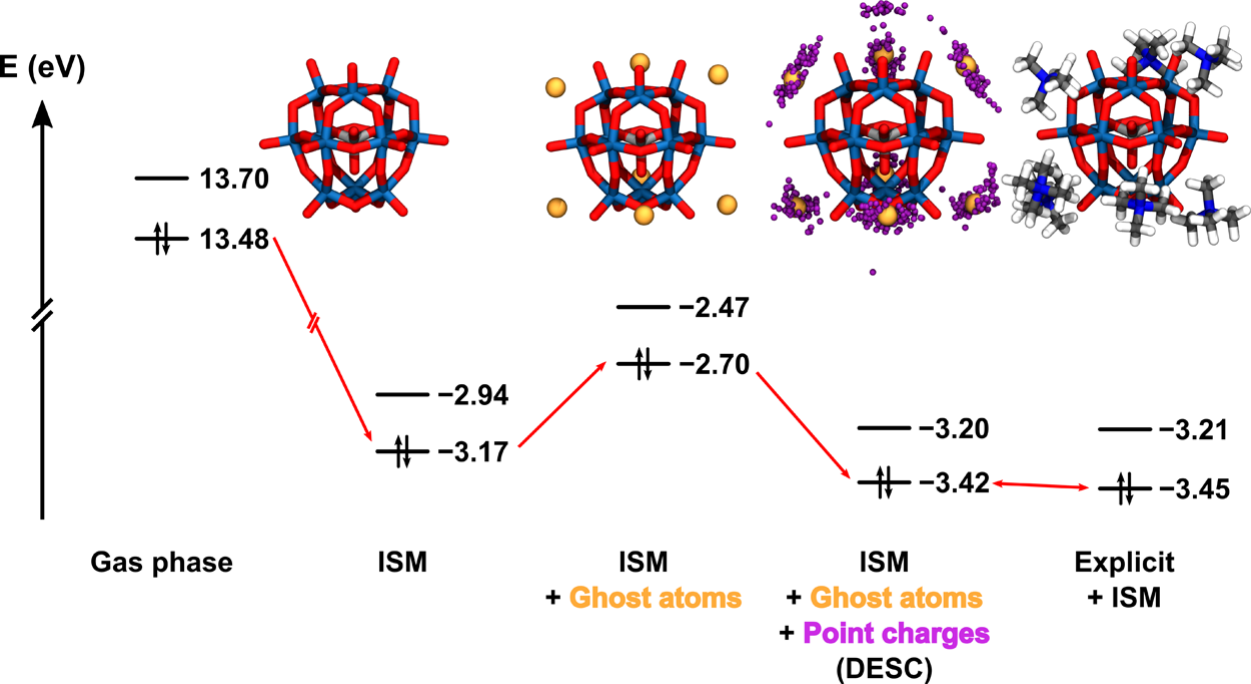
Frontier molecular orbital energies of (TMA)_7_AlK in MeCN calculated using different models. ISM corresponds to COSMO with ε = 37.5. A structural representation of the POM for each method is included. Color palette: W—blue, O—red, Al—silver, Ghost atoms—yellow, Point charges—purple, N—dark blue, C—gray, H—white.

The final output of DESC is a logfile summarizing the main results obtained during the process starting by reading a PDB file of the MD simulation. An example for (TMA)_5_AlK in MeCN, with *N* = 4.33, is also presented in the SI. In this case, where the average aggregation number is noninteger and lies within the X.22–X.78 range, DESC provides the weights of the two nearest integers in the logfile for application in each QM calculation.

### 3.5 Comparison between DESC and Explicit Model

Once the components and operating mode of DESC have been explained, let us evaluate its performance. Our benchmark values refer to the average energies of selected relevant molecular orbitals of the solute: in the present case the highest orbital of the oxo band, the HOMO, and the LUMO. In cases where the POM is fully oxidized, the first two coincide. To assess our new methodology, the orbitals obtained with DESC are compared to those from explicit model calculations, analogous to Figures 3 and S2–S3, where COSMO was compared with the explicit model. The electronic structure data obtained with the two models are presented in Figures 9 and S7–S8 for MeCN and DCM, respectively. In general, the solute molecular orbital energies obtained with DESC show semi- or quantitative agreement with the explicit model, i.e., differences no larger than 100 meV with almost no exception. Also noteworthy is the capability of DESC to differentiate between counterions, accurately reflecting the variations in the electronic structure of the POM introduced by strongly aggregated explicit counterions, which are now reproduced with a simpler model. Additionally, the energies obtained with the ISM-only model notably deviate from DESC and the explicit model deviate, with no possibility of not cation selection. Finally, execution of DESC is very fast, requiring only seconds in interactive mode for the examples presented here. This includes reading the entire PDB file, processing data, and generating the ADF input and other DESC output.

**Figure 9:**
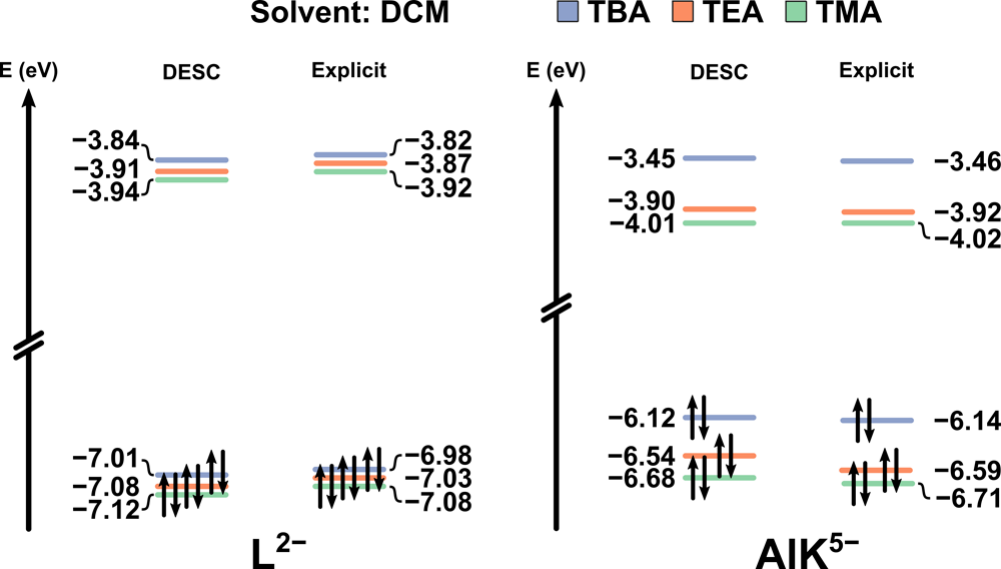
Molecular orbital diagram for **L** and **AlK** salts of TMA, TEA, and TBA in fully oxidized state, in DCM calculated following two methodologies: DESC and Explicit + ISM (average of 5 snapshots).

As shown in Figure 10, the mean absolute errors (MAEs, in meV) for DESC-based orbital energies relative to the explicit model (DESC vs explicit, orange bars) are very low, mostly below 100 meV and very frequently below 50 meV. In contrast, the quality of ISM-derived orbital energies (ISM vs explicit, purple bars) is generally much worse. Indeed, a substantial number of COSMO tests deviate as much as 500 meV, and some MAEs even exceed 1000 meV when the solvent is DCM. These results emphasize the effectiveness of the strategy and design of DESC, and bring to light the limited accuracy of a pure ISM approach for analyzing aggregated, complex systems in solution. The worst performances of DESC in the present set of calculations—such as (TEA)_7_AlK and (TBA)_7_AlK, especially in DCM—still largely outperform the homologous COSMO-based results.

**Figure 10:**
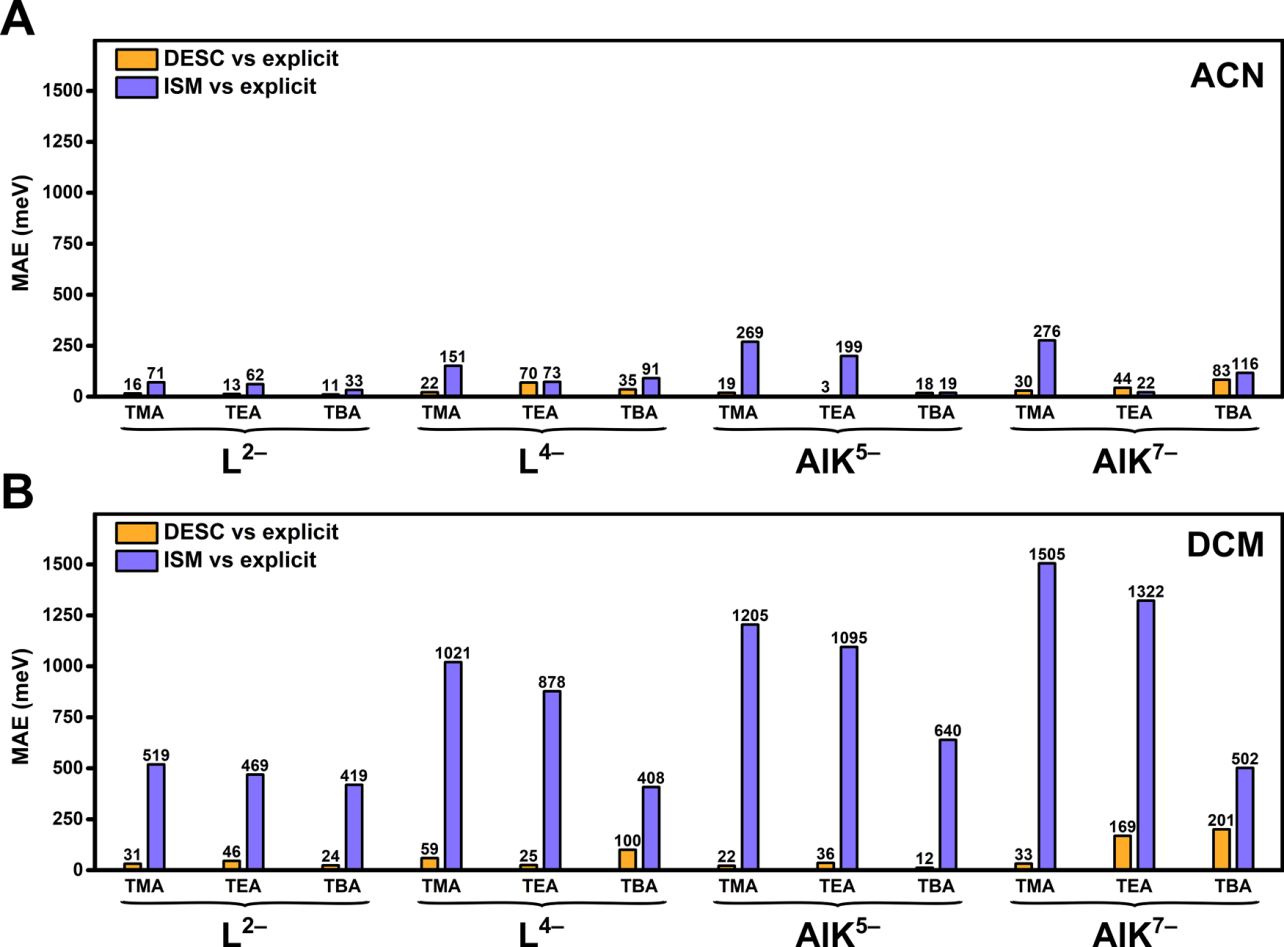
Performance of DESC for L and AlK systems. Orbital energy MAEs (vs explicit calculations) obtained with DESC (orange) and with ISM-only (purple) in (A) MeCN and (B) DCM solvents. Energy values for each group of MOs and each system are provided in Figures 3, 9, and S2, S3, S7, and S8.

Within the data presented in Figure 10, some differences are appreciable when comparing the performance of DESC in solutes with low or high charges. In the present set, fully oxidized POM states feature the lowest MAEs whereas slight discrepancies appear when calculating two-electron reduced states. The orbital differences become more pronounced, particularly in DCM, and may even lead to changes in orbital order for systems like (TMA)_7_AlK and (TEA)_7_AlK. As noted earlier, this complexity likely arises because the effects that DESC simulates in a simple approach can be nonlinear and, thus, challenging to parametrize. Considering how the relative permittivity of the medium influences electrostatic interactions, it logically follows that DESC more accurately reproduces explicit calculations in less polar solvents, where each point charge retains a higher effective value, more perceptible to the solute.

Another advantage of DESC is its remarkable reduction in QM computational times with respect to explicit model calculations, which can be difficult to converge due to the large number of electrons and the complex, eccentric wave functions (or electronic densities) that may arise. In addition, because a series of explicit calculations (5–10) is recommended for representativeness of the average energy values. Thus, one of the main goals for developing DESC was to improve convergence by focusing solely on the solute electrons (excluding those of the cosolute), thereby reducing computational times.

Computational time analysis is shown in Figure 11. All results were obtained on a single node with 32-core EPYC 7282 processors, 128 GB of memory, and 960 GB SSD disks with Infiniband. Naturally, DESC largely improves the explicit model in terms of execution time, especially for bulky counterions such as TBA, since the explicit model requires QM calculations on many more atoms (53 per cation, plus the solute). In general, the CPU times herein presented for the DESC methodology lie below 800 s. Only a few cases require up to 1000 s to execute. In contrast, most explicit calculations (for 5 snapshots) need much longer times to be completed, i.e., 3000 to 15,000 s, with some cases even more expensive. Comparing these two methodologies, the speedup factors of DESC are typically 5–20, although for the largest counterions this factor can reach 40–50.

**Figure 11:**
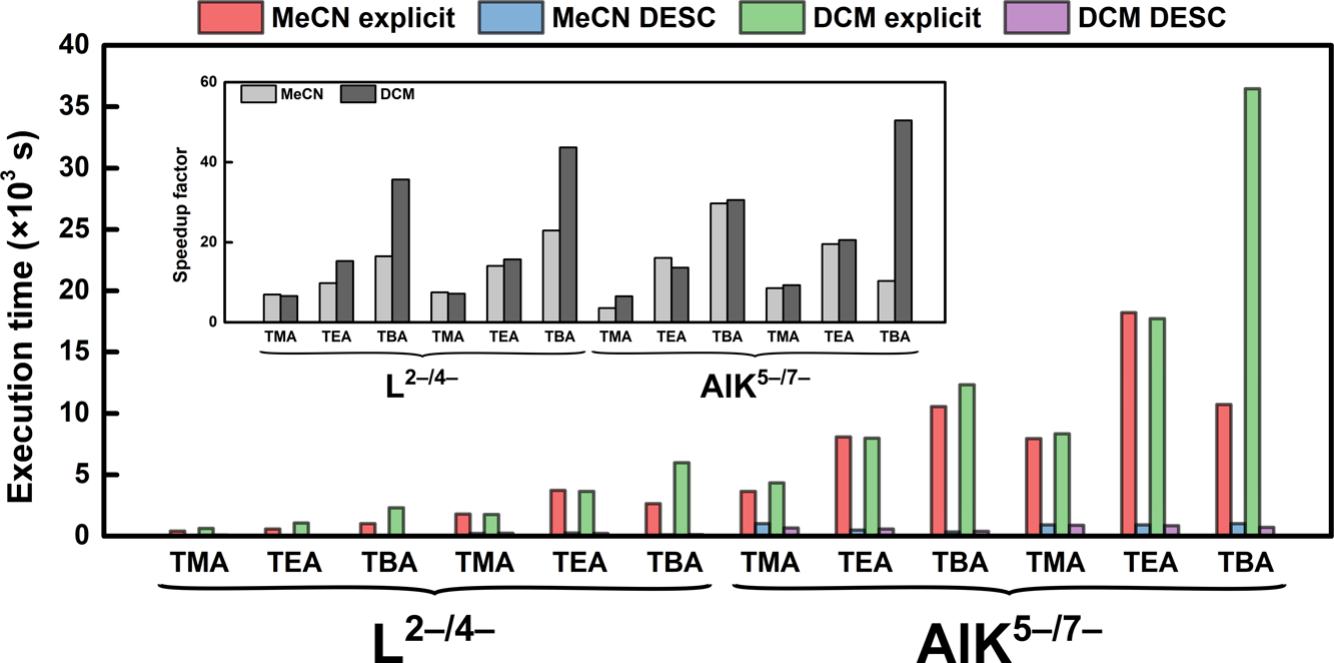
CPU times spent by DESC and explicit models for the L^2–/4–^ and AlK^5–/7–^ systems in MeCN and DCM. Inlay: speedup factor of DESC against explicit calculations. Detailed CPU times are provided in Table S2 in the SI.

What is more, the present discussion does not account for the human time required for the tasks exclusively associated with the explicit model data processing, i.e., snapshot selection from the MD trajectory, *xyz* processing, input generation of QM runs, molecular orbital energy reading and averaging. Combined, these tasks are estimated to increase the total time by ca. 30 min (1800 s) per case, unless some automatization is at work. Also, several sources of error present during file processing in the explicit model approach are ruled out from DESC.

## 4 Testing DESC in Other Systems

The effectiveness of applying DESC to POMs has been demonstrated in the previous section, in terms of speedup and accuracy when compared to calculations performed with explicit cations, which constitutes our benchmark approach. It is important to note, however, that DESC was derived by taking the Lindqvist and the aluminum-Keggin tungstate as model systems. To assess its transferability to different compounds, we herein apply DESC to other POM species and to fullerenes, as well as with other solvents.

### 4.1 Polyoxometalates with Lower Symmetry in Diverse Solvents

Additional DESC calculations were performed on POMs with other morphologies and chemical compositions than those presented in Section 3. These include the nonspherical Wells-Dawson structure, P_2_W_18_O_62_, and the vanadium-substituted Keggin phosphotungstate, PV_2_W_10_O_40_ (Figure 12a). The latter compound carries the same molecular charge (5−) and is geometrically equivalent to AlW_12_O_40_ (AlK), but shows a distinct polarized charge distribution between its tungsten and vanadium regions, leading to differences in its electronic properties. To further validate the DESC approach, we also tested its performance in two other solvents: ethanol (EtOH, ε = 24.55) and ammonia (NH_3_, ε = 16.9). Table 2 summarizes the combinations of POM-cation-solvent systems investigated and the average coordination numbers obtained (*N*), which highlight significant ion-pairing.

**Figure 12:**
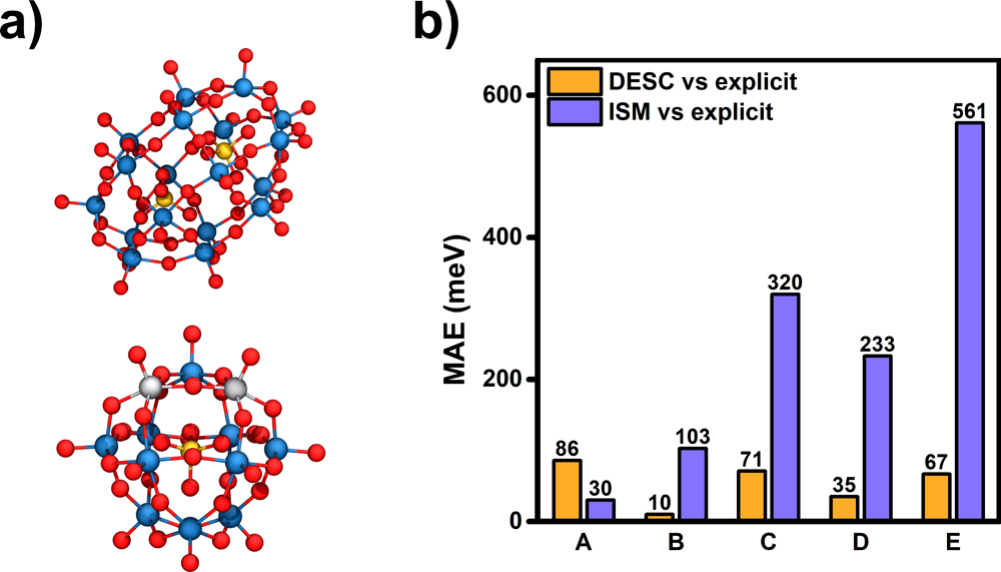
Performance of DESC in other POM systems: (a) [P_2_W_18_O_62_] (top) and [PV_2_W_10_O_40_] (bottom). Color palette: W—blue, O—red, P—yellow, V—light gray; (b) MAEs computed for orbital energies (vs explicit calculations) obtained with DESC (orange) and with ISM-only (purple) for A: (TBA)_5_[PW_10_V_2_O_40_] in MeCN, B: (TBA)_7_[PW_10_V_2_O_40_] in MeCN, C: (TMA)_6_[P_2_W_18_O_62_], D: (TEA)_7_AlK in EtOH, and E: (TEA)_7_AlK in NH_3_.

**Table 2:** Combinations of POM, Cation and Solvent Simulated for Application of DESC^a^

**POM**	cation	solvent	***N***
[PW_10_V_2_O_40_]^5–/7–^	TBA	MeCN	2.96/3.02
[P_2_W_18_O_62_]^6–^	TMA	MeCN	6.00
AlK^7–^	TEA	EtOH	7.00
AlK^7–^	TEA	NH_3_	7.00

a The average aggregation of cations around the POM is indicated.

Figure 12 compares the orbital energies calculated with DESC and ISM approaches. DESC shows consistently low MAEs in the range 10–86 meV, significantly outperforming the regular ISM approach, which errors are >200 meV in the majority of the cases, accurately reproducing the molecular orbital energies for (TBA)_5_PV_2_W_10_O_40_ in MeCN only. Despite its simplicity, eq 4 proves remarkably robust in different solvents, as evidenced by its excellent performance in ethanol and ammonia (cases D and E in Table 2, respectively). Furthermore, DESC shows transferability to nonspherical solutes, which are traditionally more challenging for such analysis (case C).

### 4.2 Ion-Pairing Effects on Reduced Fullerenes

Fullerenes represent another important class of compounds, alongside POMs, where ion-pairing can play a critical role in some of their properties and applications. Although relatively underexplored, the effect of counterions on these carbon-based molecules is significant due to their electrochemical relevance^56–58^ and their applications as electron acceptors in photoactive systems.^59–61^ Most experiments of fullerenes in solution involve supporting electrolytes (typically TBA in low-polarity or apolar solvents, such as dichloromethane, toluene or ortho-dichlorobenzene. Upon reduction, fullerenes are expected to associate strongly with cations in these media.

Although our group has focused for more than 20 years on investigating the structure and reactivity of endohedral metallofullerenes,^62–65^ in this study we apply the DESC approach to investigate the multiple reduction states of the iconic C_60_. Echegoyen and collaborators,^66^ reported the six reductions of C_60_ in a 1:5.4 (% v/v) mixture of MeCN and toluene, with TBAPF_6_ as supporting electrolyte. We have computationally mimicked these experimental conditions, focusing on the fully oxidized, singly reduced, and doubly reduced systems. For the fully oxidized state, no significant cation-fullerene association was observed, and as a result DESC and COSMO yielded identical molecular orbital energies for C_60_(ox). In contrast, for the reduced species ${C_{60}}^{-}$ and ${C_{60}}^{2-}$ , the analysis performed by DESC revealed strong ion pairing, with TBA more counterions surrounding closely the target molecule than the total negative charge of the fullerene, i.e., *N* = 2.53 and *N* = 4.72, respectively. This behavior is consistent with the low affinity of toluene for anionic fullerenes, which enhances ion-pairing. A graphical example of the TBA- ${C_{60}}^{2-}$ ion-pairing captured by DESC is shown in Figure S9. Additionally, Figure S10 shows that toluene predominates as the solvent for the reduced states, a feature that DESC identifies automatically. Consequently, all explicit counterion calculations were performed with toluene as the solvent.

The resulting frontier molecular orbital energies are shown in Table 3. DESC achieves excellent accuracy, with mean absolute errors of 47 meV for ${C_{60}}^{-}$ and 83 meV for ${C_{60}}^{2-}$ , in line to those reported for POMs. In contrast, COSMO yields very large errors (MAEs of 1920 and 3640 meV, respectively), highlighting its limitations in capturing counterion effects. Additionally, the reduction potential of ${C_{60}}^{-}$ to ${C_{60}}^{2-}$ calculated using DESC results in −1.39 V vs Fc^+/0^, in excellent agreement with the experimental value of −1.37 V vs Fc^+/0^. In comparison, the COSMO result significantly deviates with −3.26 V vs Fc^+/0^ (further details provided in the SI). These results reinforce the effectiveness of DESC in accurately capturing the influence of counterions on the electronic properties of solutes, emphasizing its potential for broader applications.

**Table 3:** Frontier Molecular Orbital Energies (in eV) for the TBA- ${C_{60}}^{-/2-}$ Systems Calculated Using Three Methods: Explicit Cations, DESC, and ISM (COSMO)

	${C_{60}}^{-}$			${C_{60}}^{2-}$		
	explicit	DESC	ISM	explicit	DESC	ISM
HOMO	–5.086	–5.135	–3.173	–5.356	–5.477	–1.756
LUMO	–4.067	–4.113	–2.149	–4.412	–4.455	–0.731

## 5 Conclusions

The occurrence of molecular aggregation in solution, including ion pairing, is an important phenomenon affecting molecular properties and chemical processes. At the computational level, accounting for this particularity is not straightforward and requires an additional effort if the accurate electronic structure of the solute is the goal. We herein present a new computational strategy that accounts for complex solution environments, such as aggregation between a solute and the cosolutes, oriented to Quantum Mechanical calculations, called Dynamic Environment in Solution by Clustering (DESC). In such cases, this approach offers greater accuracy than conventional continuum implicit solvent models (ISMs), such as COSMO or PCM. DESC incorporates the specific effects of the cosolute in a statistically averaged, general and cost-effective manner. First, the dynamic nature of the bulk—comprising solvent, cosolutes (mostly counterions), and solute—is simulated using a regular classical Molecular Dynamics trajectory. DESC extracts the averaged number of aggregated counterions and their positions, and incorporates them into a standard QM calculation as effective point charges and bulky pseudoatoms. By comparison to the explicit cations+ISM results (the benchmark data), the DESC results largely outperform the standard ISM approach if aggregation occurs. The mean absolute errors in orbital energies obtained applying DESC mostly lie below 100 meV, whereas those for ISM are much larger, in many cases between 500 and 1500 meV. The other great advantage of DESC is the substantial reduction of computational time, if compared to equivalent QM calculations with explicit cosolutes, with speedup factors ranging 5–50 for the systems analyzed. Moreover, DESC skips the tedious hands-on work of preparing and averaging multiple QM calculations, as is the case for the explicit counterion approach that needs to account for the positional variability of counterions around the solute. This is an internal task performed by DESC in a few seconds.

Overall, the model presented here efficiently incorporates counterion mobility with a drastic reduction of computational effort and distinguishes the effects of different counterions, as evidenced by changes in the solute molecular orbital energies as the cosolute varies. This strategy was initially developed and tested with highly symmetrical polyoxometalate systems as target solutes but, in principle, there are no limitations in this aspect. Hence, any combination of solute, cosolute and solvent can be analyzed and processed by DESC. Tests conducted on different systems, such as nonsymmetrical POMs and a fullerene, demonstrate that the methodology is robust also for solutes of other nature, clearly outperforming classical ISMs.

Further work on this model is ongoing in our group to generalize the current performance, especially when strong ion-pairing occurs, which might originate in important changes in the bulk solvent’s relative permittivity. Classical electrolyte theories, such as Debye–Hückel theory or Born and Poisson–Boltzmann solvation models, are valid only in sufficiently dilute solutions,^67^ where ion–ion correlations are negligible. Future improvements to the model could involve addressing this issue by refining the treatment of point charges, potentially employing a screened Coulomb potential, such as a Yukawa function or similar approach.^68^ Yet another variation to the present version will include accounting correctly for the effects of solvent mixtures.

The associated DESC Python code is open-access and can be freely installed.

## Supplementary Material



## Notes

DFT-optimized and DESC-generated interatomic distances for AlK; CPU times for DESC and explicit calculations; solute-counterion RDFs; pre-DESC reorientation step for MD trajectories; molecular orbital energies obtained with DESC vs the number of frames taken from the MD trajectory; comparisons of molecular orbital energies in DCM and MeCN solvents; additional text: DESC-related files (main input, logfile, generated ADF input); and redox information related to the C_60_ system (PDF)
