# Supplementary material for: DESC: An Automated Strategy to Efficiently Account for Dynamic Environment Effects in Solution
Source: J Chem Theory Comput. 2025 Feb 28;21(5):2472–86. doi: 10.1021/acs.jctc.5c00002 (PMC13192329; doi:10.1021/acs.jctc.5c00002)
Supplement: Supplementary file 1 [file ct5c00002_si_001.pdf]

Supporting Information: DESC: An Automated Strategy to Efficiently Account for Dynamic Environment Effects in Solution

Albert Masip-Sánchez, Josep M. Poblet, and Xavier López*

Departament de Química Física i Inorgànica, Universitat Rovira i Virgili (URV), Marcel·lí Domingo 1, 43007 Tarragona (Spain)

E-mail: javier.lopez@urv.cat

Table S1: Bond distances in Å for the different types of bonds in an **AlK**. O_c refers to the central oxygen in AlO_4, O_t to the terminal oxygens, and O_b to the bridging oxygens.

**Bond**	**Optimized**	**DESC**
Al-O_c	1.771	1.774
W-O_t	1.761	1.754–1.758
W-O_b	1.948–1.966	1.946–1.969
W-O_c	2.303	2.302

Table S2: CPU times spent, in h:mm:ss, applying DESC and the explicit model, for $L^{2-/4-}$ and $AlK^{5-/7-}$ systems in MeCN and DCM. The speedup factor of DESC vs explicit calculations is shown in each case.

	TMA		TEA		TBA	
**POM**	**Explicit**	**DESC**	**Explicit**	**DESC**	**Explicit**	**DESC**
L^{2-} (MeCN)	0:07:09	0:01:02	0:09:53	0:01:00	0:17:19	0:01:03
AlK^{5-} (MeCN)	1:00:58	0:17:07	2:14:54	0:08:23	2:56:04	0:05:55
Speedup		×5		×13		×23
L^{4-} (MeCN)	0:30:10	0:04:01	1:02:16	1:02:16	0:44:18	0:01:56
AlK^{7-} (MeCN)	2:12:33	0:15:32	5:03:41	5:03:41	2:59:02	0:17:18
Speedup		×8		×17		×17
L^{2-} (DCM)	0:10:40	0:01:37	0:18:11	0:01:11	0:38:35	0:01:05
AlK^{5-} (DCM)	1:12:34	0:11:09	2:13:20	0:09:46	3:25:47	0:06:44
Speedup		×7		×15		×33
L^{4-} (DCM)	0:29:30	0:04:07	1:00:49	0:03:52	1:39:58	0:02:17
AlK^{7-} (DCM)	2:19:13	0:14:59	4:55:25	0:16:02	10:07:45	0:12:03
Speedup		×8		×17		×47

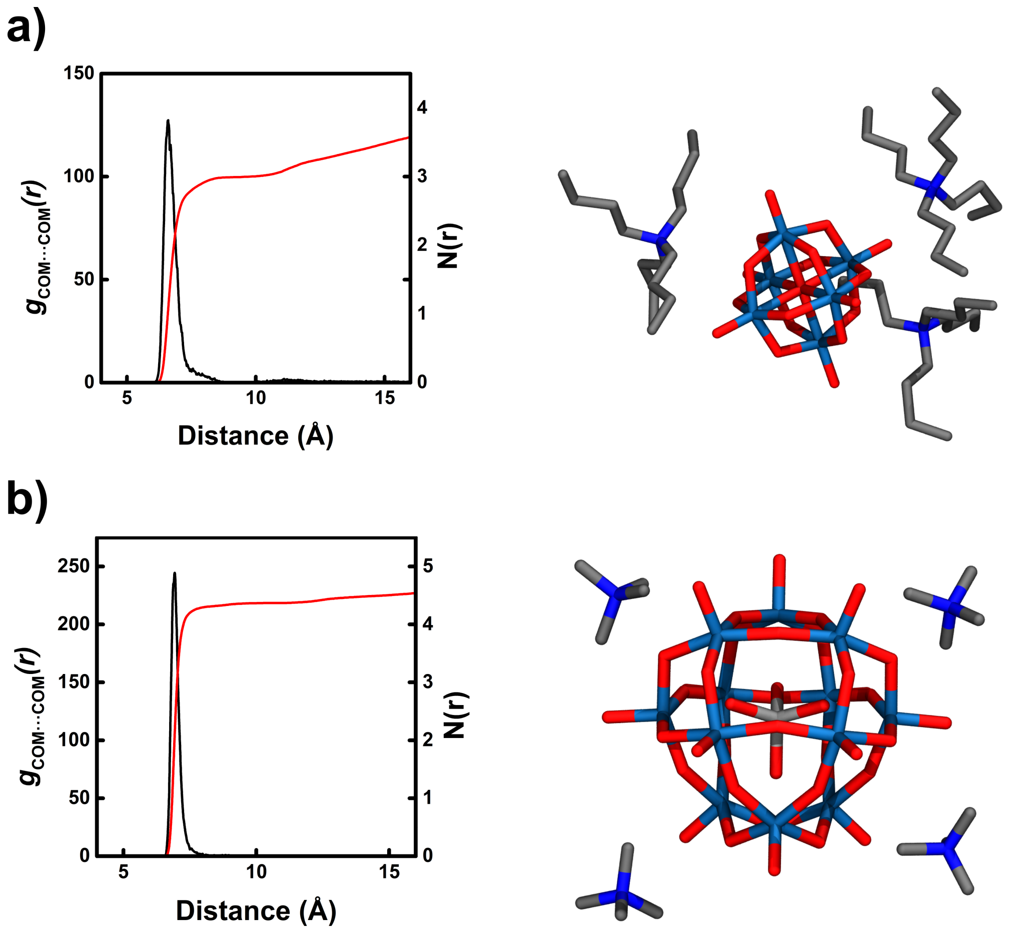

Figure S1: Radial Distribution Function (RDF) (black line), and the associated integration vs distance (red line), between the centers of mass of the POM and the cations. Case a) $(TMA)_5AlK$ in MeCN, case b) $(TBA)_4L$ in DCM. Data taken from the last 10 ns of the trajectory, sampled every 1 ps. A representative snapshot is included, showing the high level of aggregation. Hydrogen atoms are omitted for clarity. Color palette: W - blue, O - red, Al - silver, N - dark blue, C - gray.

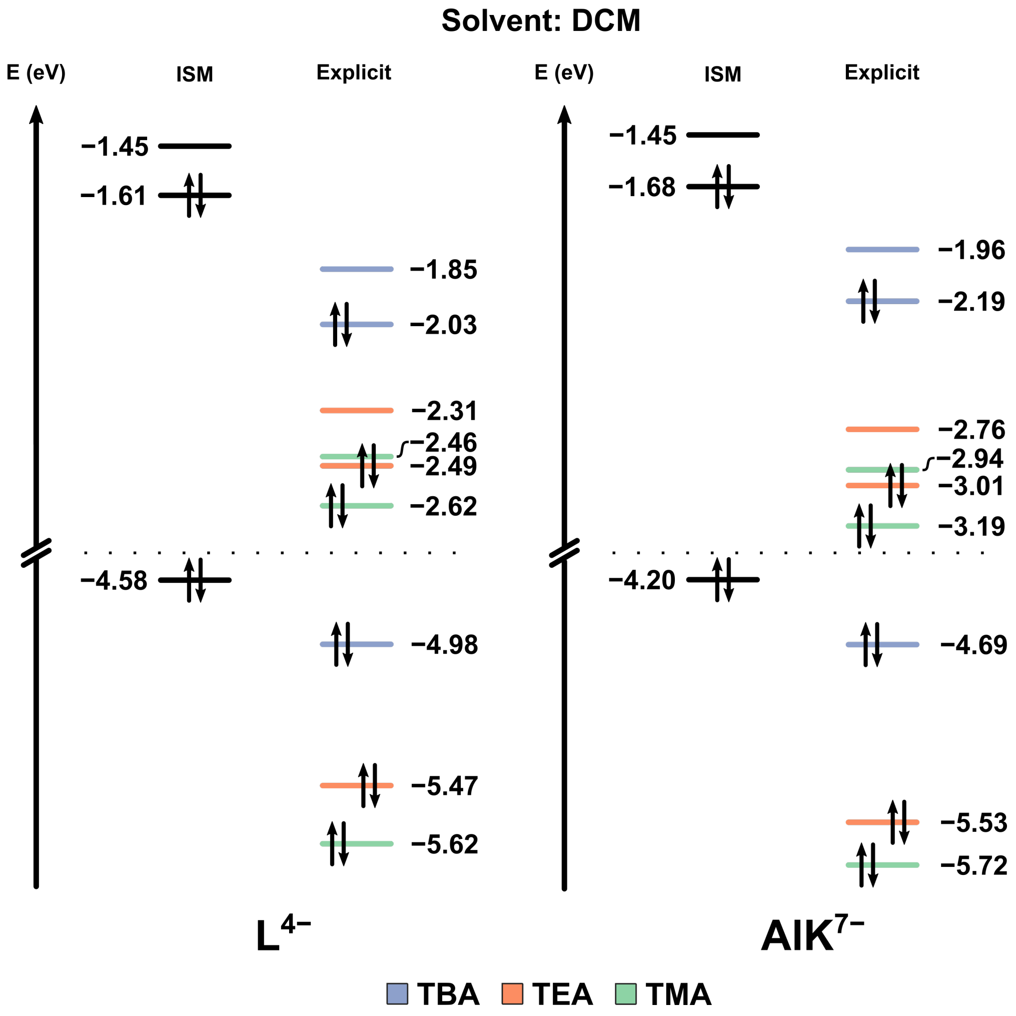

Figure S2: Molecular orbital diagram for L and AlK salts of TMA, TEA and TBA in 2e-reduced state in DCM calculated following two methodologies: ISM (COSMO, $\varepsilon = 8.9$) and Explicit+ISM (average of 5 snapshots).

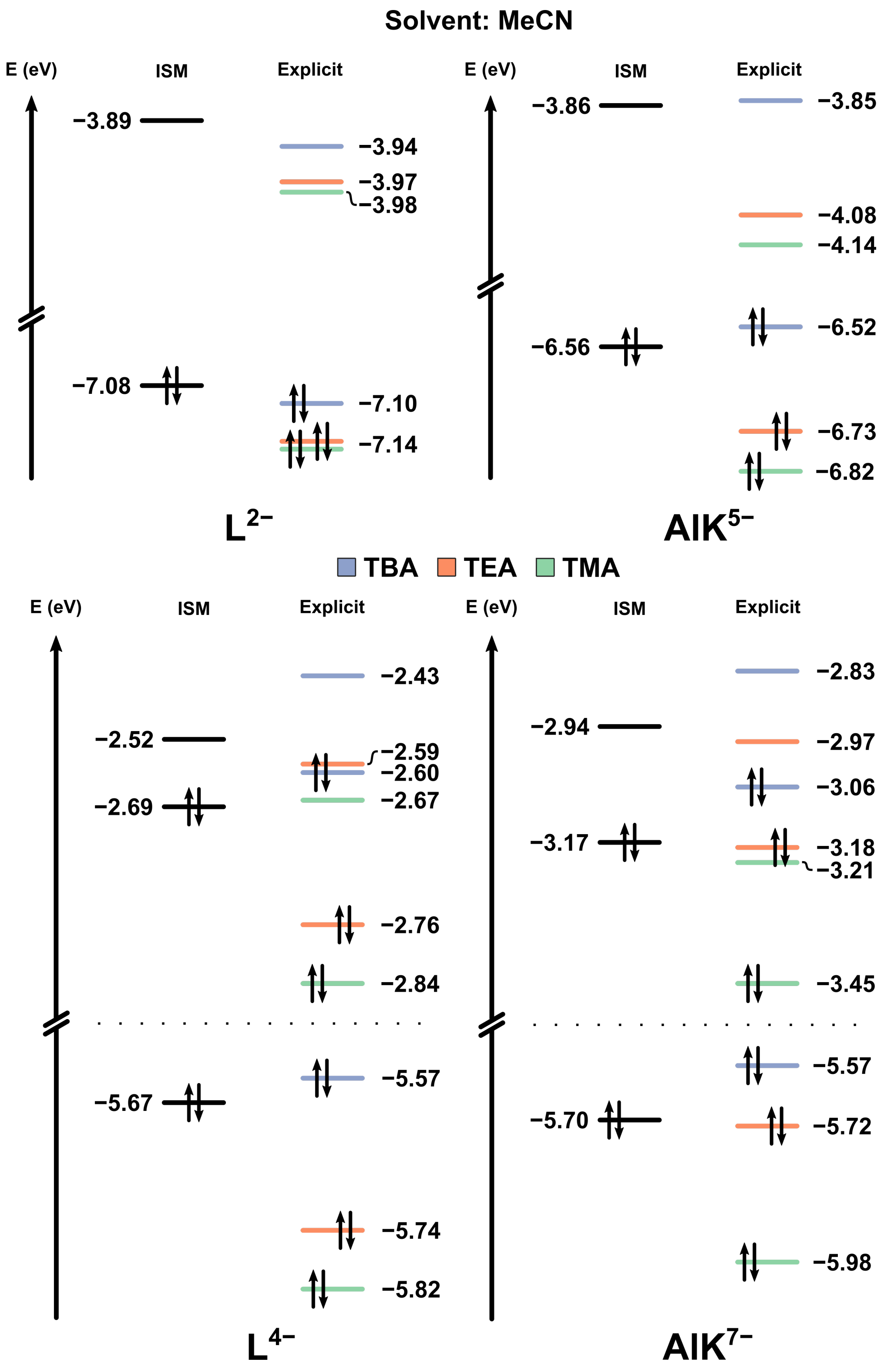

Figure S3: Molecular orbital diagram for L and AlK salts of TMA, TEA and TBA in fully oxidized and 2e-reduced states in MeCN calculated following two methodologies: ISM (COSMO, $\varepsilon = 8.9$) and Explicit+ISM (average of 5 snapshots).

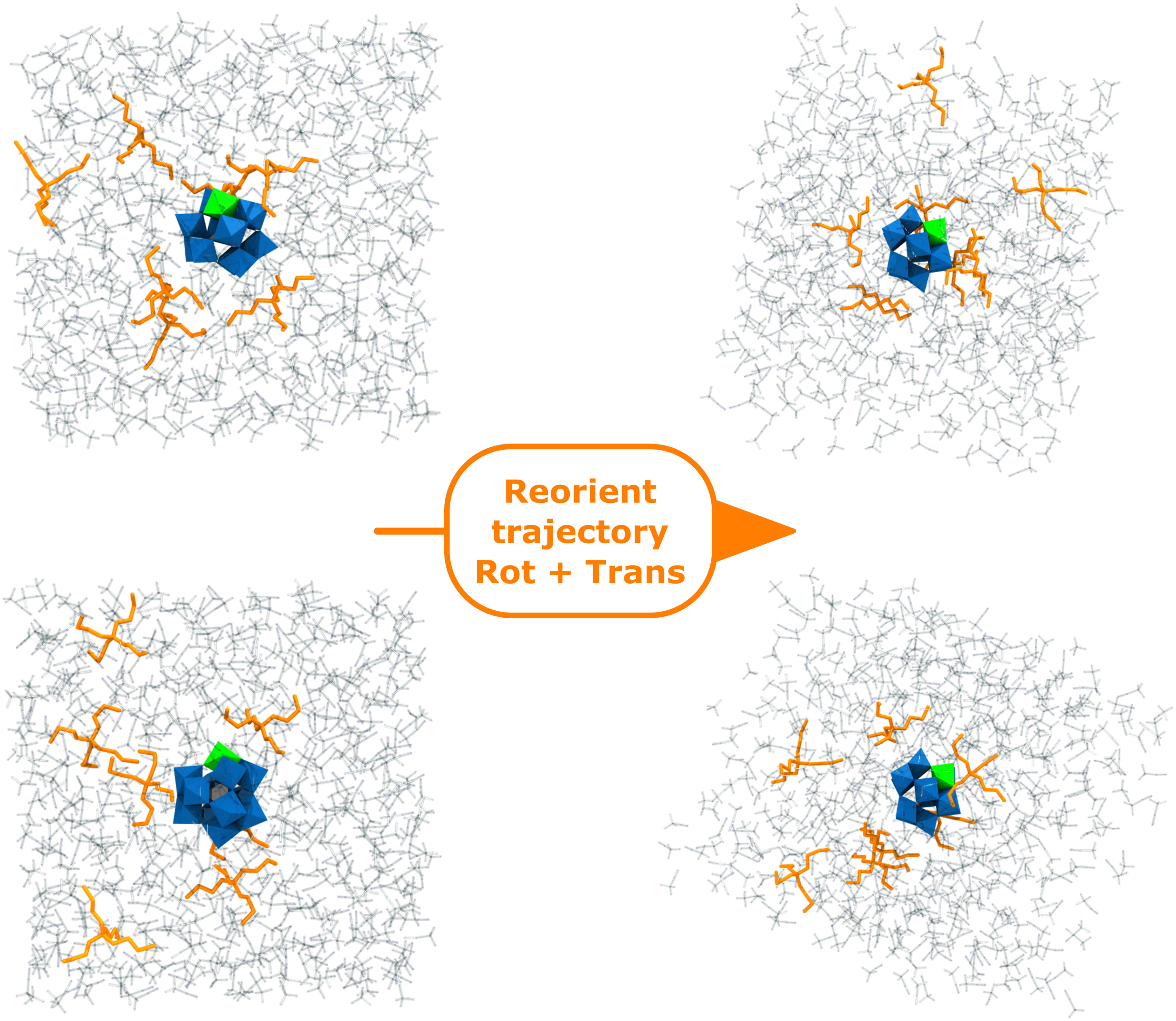

Figure S4: Functioning of the necessary reorientation step for a POM-cation MD simulation. The reorientation of the POM is shown by marking in green one position as reference. In the simulation (left frames) it has freedom of movement whereas, after reorientation (right frames), its position has been fixed and the environment adapts and moves around it. Color palette: WO_6 - blue and green polyhedra, Al - silver, TBA - orange, MeCN - gray.

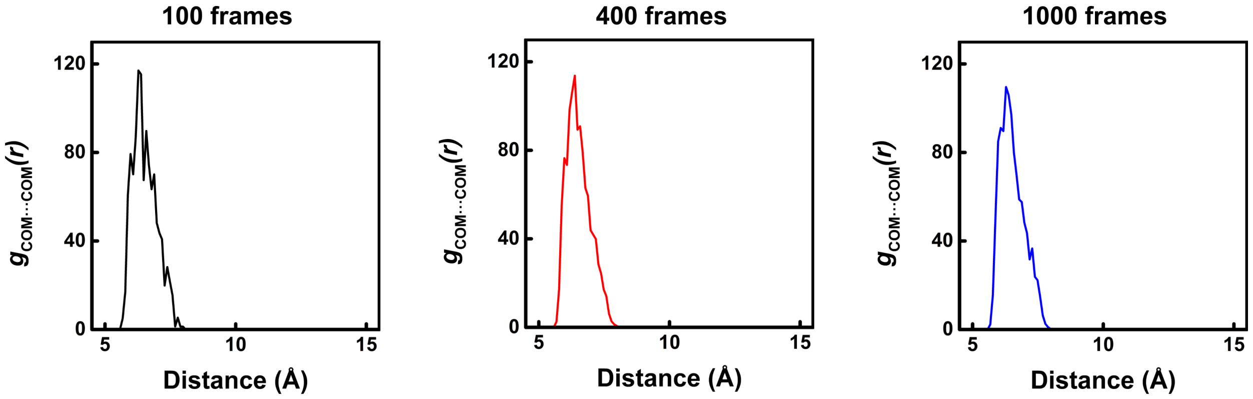

Figure S5: Radial distribution functions computed for a (TMA)L salt, varying the number of frames selected from the trajectory. An increasing number of frames improves, to a certain limit, the resolution of the $g(r)$ function. The split of 400 frames in DESC suffices for a good quality.

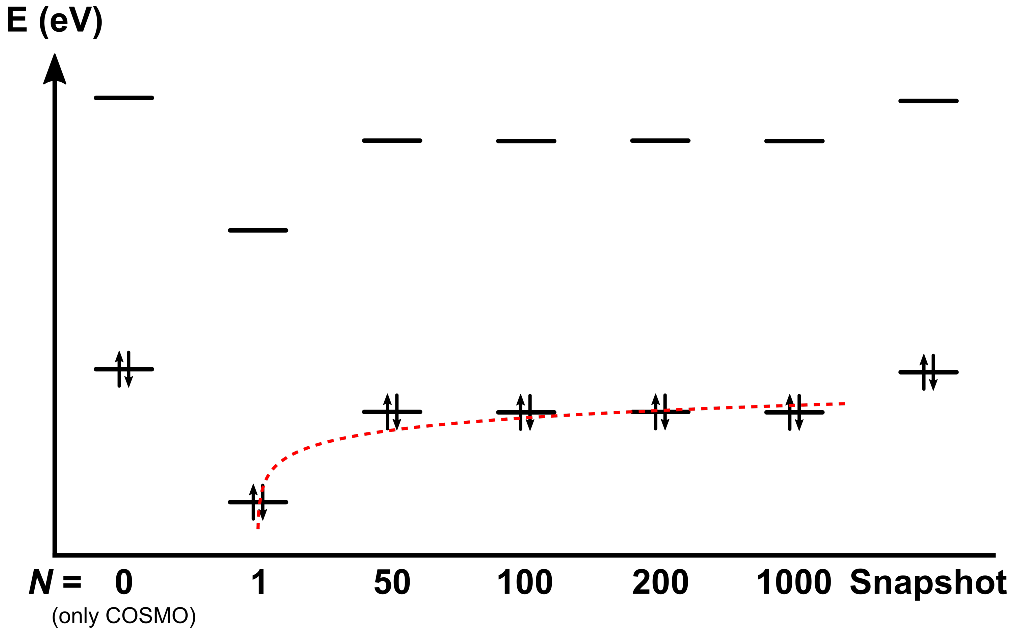

Figure S6: Schematic view of the energies of the frontier molecular orbitals of a L^{2-} aggregate, as a function of the number of frames selected by DESC in the second split. The energies quickly converge to a constant value around $N = 50$. Selecting $N = 100$ makes DESC totally robust.

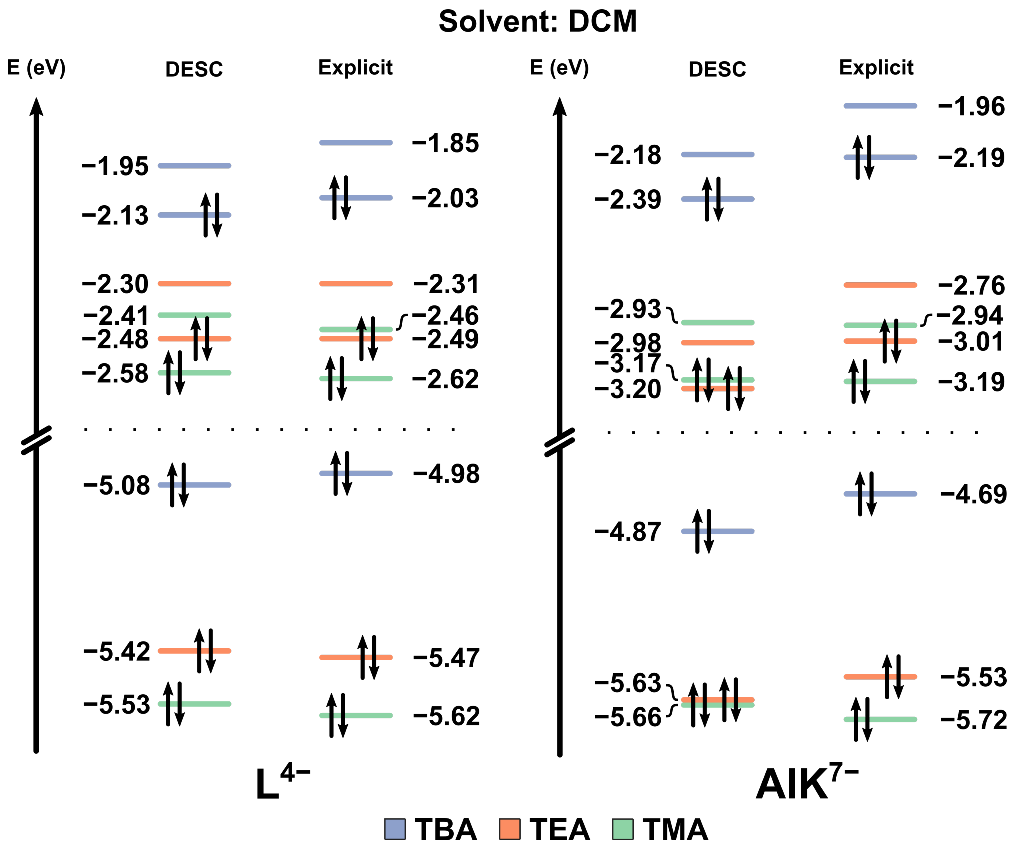

Figure S7: Molecular orbital diagram for L and AlK salts of TMA, TEA and TBA in 2e-reduced state in DCM calculated following two methodologies: DESC and Explicit+ISM (average of 5 snapshots).

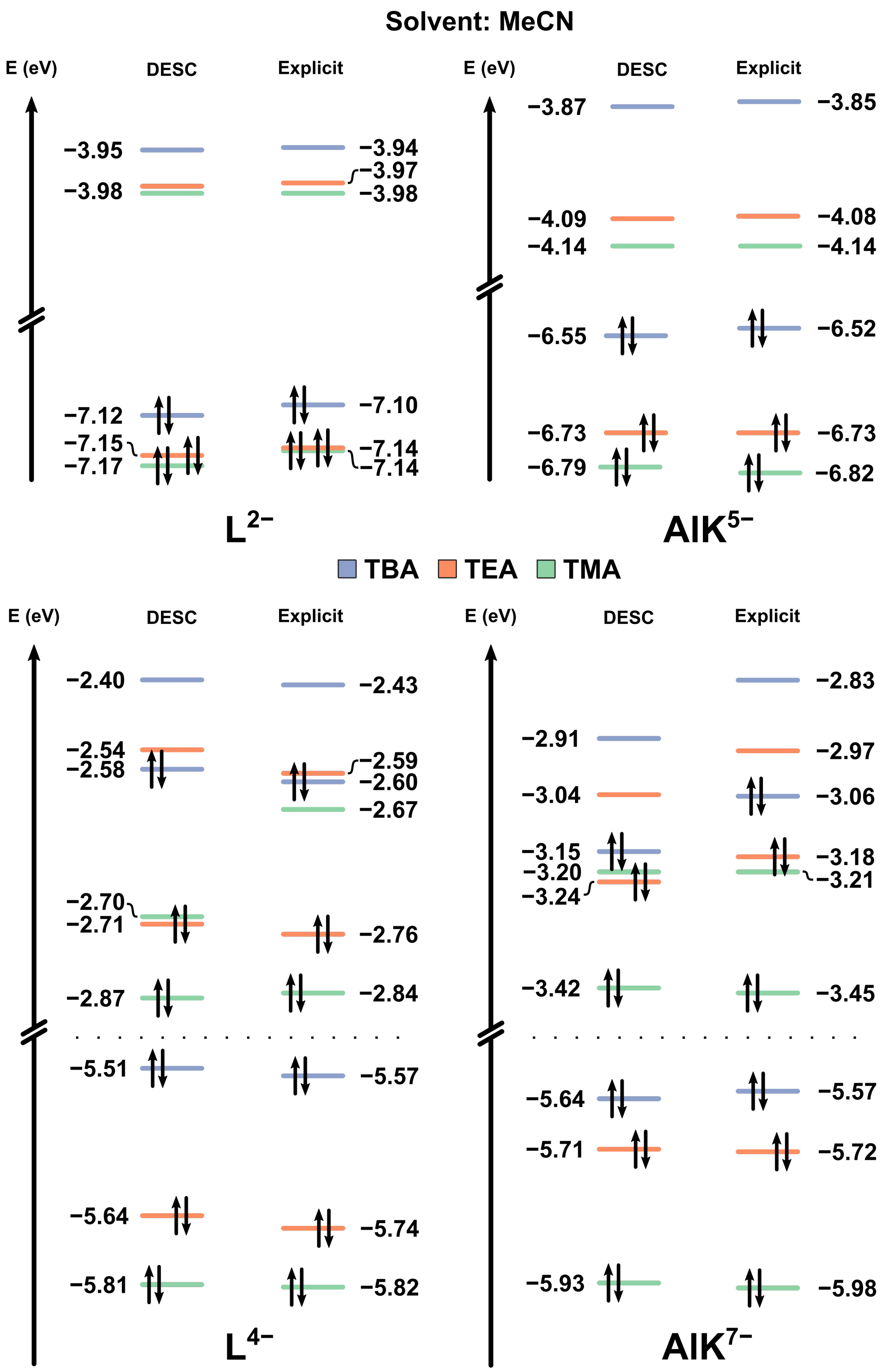

Figure S8: Molecular orbital diagram for L and AlK salts of TMA, TEA and TBA in fully oxidized and 2e-reduced states in MeCN calculated following two methodologies: DESC and Explicit+ISM (average of 5 snapshots).

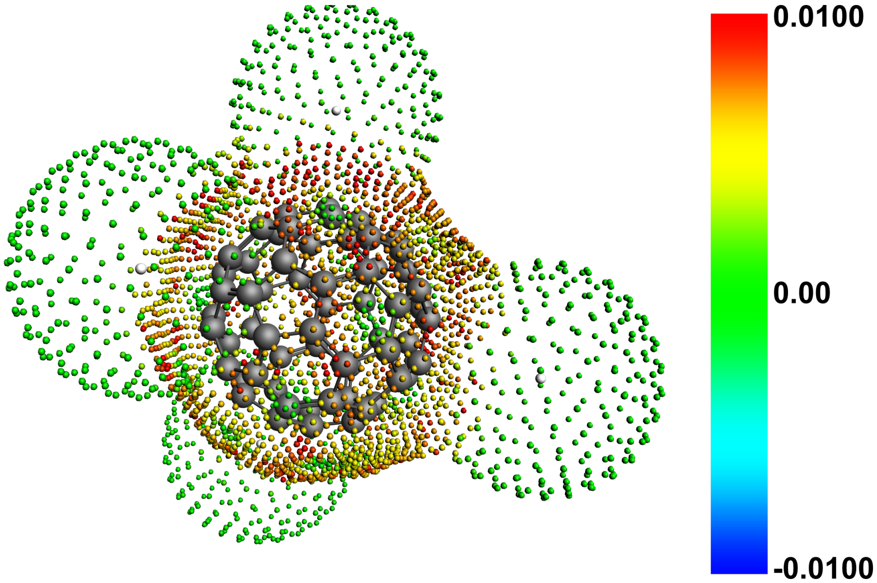

Figure S9: COSMO charge density surfaces for TBA-$C_{60}{}^{2-}$ corresponding to the DESC approach. A color bar indicating charge density is included.

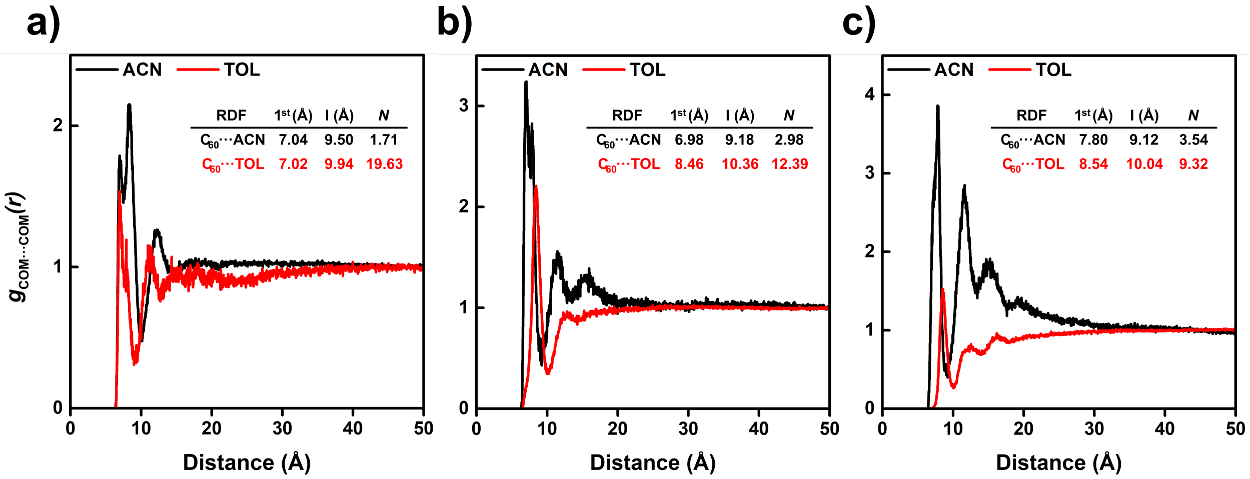

Figure S10: Radial Distribution Function (RDF) between the centers of mass of the C_{60} and the solvents, acetonitrile (ACN, black line) and toluene (TOL, red line) for the three reduction states analyzed for the fullerene: a) fully oxidized, b) one-electron reduced, and c) two-electron reduced. Data taken from the last 10 ns of the trajectory, sampled every 1 ps.

Input file of DESC

```
#### TRAJECTORY

# Trajectory filename

  traj        = traj.pdb

# Number of frames to save

  frames      = 400

# Reference residue name (solute)

  ref         = KEG

#### RESIDUES

# Atom name, residue name and charge of each residue

# in case of multiple residues, introduce them

  separated by a blank space

  atname       = N1

  resname      = TMA

  qform        = 1
```

Ready-to-run ADF input file

```
  ATOMS

  W     23.994   39.111   36.106

  W     24.788   37.158   33.460

  W     24.589   40.500   33.081

  W     27.599   38.963   32.941

  W     26.804   40.917   35.585

  W     27.005   37.576   35.965

  O     22.672   39.168   37.266

  O     25.128   40.603   36.672

  O     23.349   40.260   34.662
```

```
O      23.513   37.594   34.971
O      25.288   37.925   36.976
O      25.958   36.378   34.825
O      24.031   38.705   32.529
O      23.698   41.575   32.018
O      24.042   35.773   32.683
O      25.796   39.036   34.524
O      28.921   38.914   31.781
O      26.466   37.472   32.376
O      28.240   37.806   34.380
O      28.080   40.481   34.077
O      26.307   40.150   32.073
O      25.636   41.699   34.221
O      27.562   39.371   36.517
O      27.896   36.498   37.028
O      27.551   42.298   36.370
Gh.H  20.140   39.440   33.770  adf.R=1.868
Gh.H  31.400   39.690   36.130  adf.R=1.868
Gh.H  26.170   39.320   28.690  adf.R=1.868
Gh.H  23.860   34.550   37.610  adf.R=1.868
END
ElectrostaticEmbedding
  MultipolePotential
    Coordinates
    x1     y1     z1     qfitt
    x2     y2     z2     qfitt
    x3     y3     z3     qfitt
```

```
    ...
    ...
    End
  End
End
```

Magnitude of point charges

The first logical approximation for the magnitude of the point charges is to use the formal charge of the entire cation, $q = +1$ in these examples. However, as described in the main text, this leads to over-stabilization of the solute because the point charges do not account for the presence of the implicit solvent. Using the orbital energies of the explicit model for the (TMA)L system in MeCN as a reference, these are -5.82, -2.84, and -2.67 eV for the oxo band, the HOMO, and the LUMO, respectively. DESC reproduces these energies with very low MAEs, but if the formal charge is used instead of the q_{fitt}, the calculated energies become -14.72, -11.72, and -11.56 eV, respectively.

Logfile of DESC

```
Trajectory analysis done!
Atom Name: N1
Residue: TMA
Solvent: Acetonitrile
Integration distance: I = 8.582
Highest peak distance: 1st = 6.976
v-Rg of TMA: 1.869 ± 0.001
Average number of counterions per snapshot: N = 4.329
N=4 weight: 0.671, N=5 weight: 0.329
Execution time: 38 seconds
```

Reduction of C_{60} in toluene

The reduction potential associated with the electron transfer process is calculated employing Nernst's equation (Equation 1).

$$E(\text{red}) = -\frac{\Delta E}{nF} - E(\text{Fc}^{+/0}), \tag{1}$$

where ΔE is the change in electronic energy between the reduced and oxidized states, n is the number of electrons involved in the process, n=1 in the present case, and F is Faraday's constant. The reported absolute energy for the $\text{Fc}^{+/0}$ electrode in toluene is $E_{\text{Fc}^{+/0}} = 5.214$ eV, computed based on the reference for $\text{DmFc}^{+/0}$ vs. $\text{Fc}^{+/0}$ in toluene[S1] and $\text{Fc}^{+/0}$ vs. SHE in MeCN.[S2] For this calculation, alterations within the coordination sphere throughout the transition from ${C_{60}}^{-}$ to ${C_{60}}^{2-}$ were not accounted for.

References

(S1) Barrière, F.; Geiger, W. E. Use of weakly coordinating anions to develop an integrated approach to the tuning of Δ E 1/2 values by medium effects. *Journal of the American Chemical Society* **2006**, *128*, 3980–3989.

(S2) Pavlishchuk, V. V.; Addison, A. W. Conversion constants for redox potentials measured versus different reference electrodes in acetonitrile solutions at 25 C. *Inorganica Chimica Acta* **2000**, *298*, 97–102.
